# A Convenient One-Pot Synthesis of Novel Benzimidazole–Thiazinone Derivatives and Their Antimicrobial Activity

**DOI:** 10.3390/antibiotics13121155

**Published:** 2024-12-02

**Authors:** Sabahat Samreen, Asghar Ali, Saiema Ahmedi, Mohammad Raghib, Anzarul Haque, Nikhat Manzoor, Afzal Hussain, Mohammad Abid, Afreen Inam

**Affiliations:** 1 Medicinal Chemistry Laboratory, Department of Chemistry, Jamia Millia Islamia, Jamia Nagar, New Delhi 110025, India; sam17saba@gmail.com (S.S.); mohdraghib007@gmail.com (M.R.); 2 Department of Biochemistry, School of Chemical and Life Sciences (SCLS), Jamia Hamdard, New Delhi 110062, India; asgharalijmi@gmail.com; 3 Department of Biosciences, Jamia Millia Islamia, Jamia Nagar, New Delhi 110025, India; saiemaahmedi1995@gmail.com (S.A.); nmanzoor@jmi.ac.in (N.M.); 4 Central Laboratories Unit, Qatar University, Doha 2713, Qatar; anzarul.h@qu.edu.qa; 5 Department of Pharmacognosy, College of Pharmacy, King Saud University, Riyadh 11451, Saudi Arabia; afihussain@ksu.edu.sa

**Keywords:** thiazinone, TBTU, antibacterial, antifungal, *Candida* species, synergetic effect

## Abstract

**Background:** The increasing prevalence of antimicrobial resistant highlights the urgent need for the new therapeutic agents. This study aimed to design and synthesize fused tricyclic benzimidazole–thiazinone derivatives (**CS1**–**CS10**) through a convenient method and evaluate their antimicrobial activity against various microorganisms. **Methods:** A series of fused tricyclic benzimidazole–thiazinone derivatives was rationally designed and synthesized in one pot by the reaction between trans substituted acrylic acids and 1*H*-benzo[d]imidazole-2-thiol using coupling reagent TBTU (2-(1*H*-benzotriazol-1-yl)-1,1,3,3-tetramethyluronium tetrafluoroborate). The structure of these compounds was confirmed through various spectroscopic techniques like IR, ^1^H and ^13^C NMR, the DEPT and 2D-HMQC NMR techniques were also performed to confirm the relation of both carbon and proton. Further, the compounds were in vitro evaluated for their effectiveness against the *Candida* species and a panel of standard bacterial isolates. **Results:** The synthesized compounds showed moderate antimicrobial activity. Among all of the compounds, **CS4** exhibited potent inhibition against *Pseudomonas aeruginosa* and *Escherichia coli* at 256 and 512 μg/mL concentrations, respectively. Additional research indicated that compound **CS4** demonstrated a synergistic effect after combining with the standard antibacterial drug ciprofloxacin. **Conclusions:** These results suggest that **CS4** is the best-synthesized antibacterial agent particularly in combination therapies. These findings highlight its promise for further development as a novel antibacterial agent.

## 1. Introduction

Communicable diseases pose a significant global challenge, resulting in substantial harm to both the well-being of individuals and the economies of impoverished and developing nations. According to the World Health Organization (WHO), over 3.5 million fatalities occur annually as a result of infectious diseases [1]. The global efficacy of medical therapies is jeopardized by the emergence of resistance. The improper or excessive utilization of antibiotics and antifungal medications contributes to the emergence of resistant strains, posing challenges in treating infections [2]. Finding an equilibrium between utilizing the advantageous functions of microorganisms in the environment and mitigating the potential hazards linked to diseases is essential for the long-term viability of ecosystems and for worldwide public health. In 2019, the Centers for Disease Control and Prevention (CDC) released a report about microbial infection and it was estimated that, in the United States, over 2.8 million people carried bacteria or fungi that are resistant to antibiotics. Additionally, the report stated that there were approximately 35,000 deaths annually as a result of these infections [3]. As per the 2022 CDC report, the COVID-19 pandemic exacerbated this scenario, leading to an increased prevalence of infection [4]. Thus, to combat this growing threat, ongoing research and collaborative efforts are essential to understand the dynamics of these diseases and to set up various strategies for the development of novel therapeutic agents.

Heterocyclic compounds exhibit substantial biological potential and serve as the nexus between chemistry and biology. Over 90% of novel medications have heterocyclic themes [5,6]. Sulfur-and-nitrogen-containing heterocyclic compounds such as thiazole [7], thienopyrimidine [8], thiomorpholine [9], thiazine and benzothiazines heterocycles exhibit distinct properties as biologically active agents and have captivated researchers for many years due to their historical significance in the field of organic synthesis [10]. Recently, thiazine has captured the interest of scientists as a significant structural motif in medicinal chemistry [11]. It is composed of sulfur (S) and nitrogen (N) atoms with four carbon atoms arranged in a six-membered ring and is categorized into three groups depending on the positions of the N and S atoms in the ring: 1,2-thiazine, 1,3-thiazine, and 1,4-thiazine [12]. Moreover, benzimidazole is described as a fused bicyclic heteroaromatic compound, composed of a benzene ring and imidazole ring. The presence of a nitrogen atom makes the benzimidazole ring more electron-rich, explaining why it can donate or accept electrons and also easily form weak interactions with different biological targets [13]. Both motifs have garnered significant attention due to their promising therapeutic potential, serving as antibacterial, antifungal, anti-inflammatory, antiviral, anticancer, anticonvulsant, antihistamine, and antidepressant agents [14,15].

**Figure 1:**
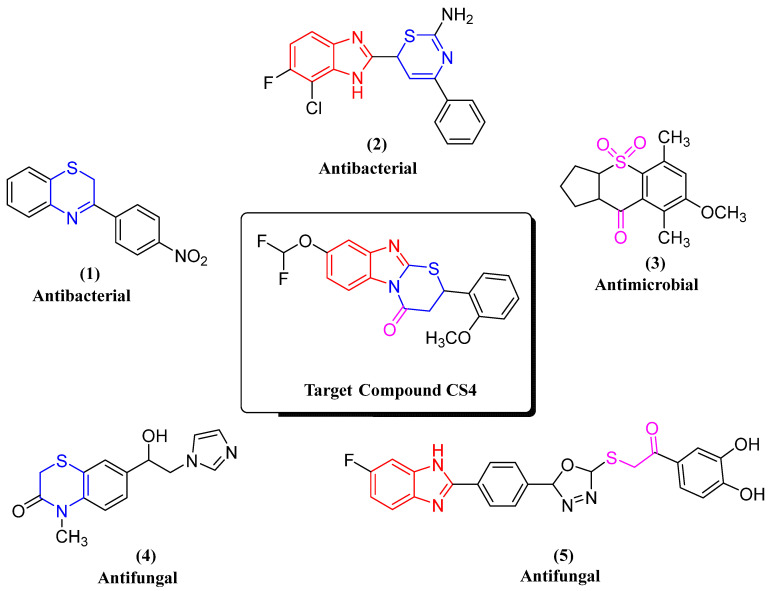
Rational design of the proposed compound [23,24,25,26,27].

The direct production of amide linkages by the interaction of an acid and an amine employing a coupling reagent is a well-established and preferable method in organic chemistry. Generally, 25% of pharmacophores contain an amide linkage [16]. Coupling reagents are used to facilitate the necessary link between two reagents that would not naturally react under mild conditions. Some common coupling reagents are dicyclohexylcarbodiimide (DCC), 1-ethyl-3-(3-dimethylaminopropyl)carbodiimide (EDC), carbonyldiimidazole (CDI), 1-[*Bis*(dimethylamino)methylene]-1*H*-1,2,3-triazolo [4,5-*b*]pyridinium 3-oxid hexafluorophosphate (HATU), *O*-benzotriazole-*N,N,N’,N’*-tetramethyl-uronium hexafluorophosphate (HBTU), 2-(1*H*-benzotriazol-1-yl)-1,1,3,3-tetramethyluronium tetrafluoroborate (TBTU), etc., which vary in their mechanisms and application conditions [17,18]. TBTU is a benzotriazole coupling reagent and TBTU-based coupling reactions are documented in various scientific publications. Bianca C. Perez et al. published a study discussing the formation of a series of novel heterocycle−cinnamic conjugates by combining cinnamic acids and 8-aminoquinoline utilizing TBTU as a catalyst [19]. Further, a study has documented a reaction dealing with the formation of the amide bond of *N*-acetyl-L-phenylalanine utilizing TBTU as a means of synthesizing 2-(*N*-acetyl)-L-phenylalanylamido-2-deoxy-D-glucose, which exhibits anti-inflammatory properties [20]. Additionally, a comparable compound, 2-aryl-2,3-dihydro-4*H*-[1,3]thiazino[3,2,*a*]benzimidazoil-4-one, was synthesized using DCC [21]. However, the extraction of the compound proved to be a complex process due to the formation of a water-insoluble byproduct called dicyclohexylurea (DCU) [22]. These studies have aided the discovery of an alternative pathway for synthesizing benzimidazole–thiazinone derivatives by employing TBTU. Further, the structural resemblance of synthesized derivatives with several physiologically active heterocycles containing nitrogen and sulfur, exhibiting antifungal and antibacterial properties, is shown in Figure 1.

## 2. Results and Discussion

### 2.1. Chemistry

**Scheme 1:**
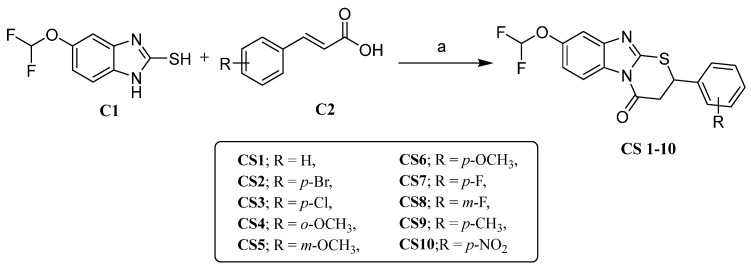
Reagents and conditions: (**a**) TBTU, DIPEA, dry DMF, stirred at room temperature for 36 h.

**Scheme 2:**
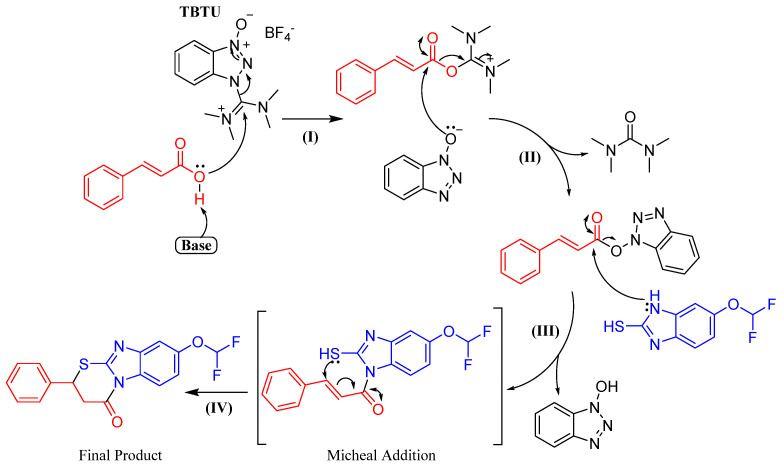
Proposed mechanism for the formation of benzimidazole–thiazinone derivatives.

The detailed protocol for the synthesis of derivatives (**CS1–CS10**) is given in Scheme 1. Substituted benzimidazole–thiazinone derivatives were synthesized via a single-step multicomponent system utilizing TBTU at room temperature. A hypothetical mechanism of reaction [20] is given in Scheme 2. A coupling reaction was performed between (difluoromethoxy)-1H-benzo[d]imidazole-2-thiol **C1** and different substituted trans acrylic acids **C2** in the presence of coupling reagent TBTU and base *N*,*N*-diisopropylethylamine (DIPEA) using dry N,N-dimethylformamide (DMF) as a solvent. In the first step, TBTU facilitated the formation of an O-acylisourea intermediate followed by the formation of an active ester with the coupling reagent attached in the second step. In the third step, nucleophilic attack detached the benzotriazole group to generate the supposed intermediate, (*E*)-1-(5-(difluoromethoxy)-2-mercapto-1H-benzo[d]imidazol-1-yl)-3-phenylprop-2-en-1-one. Then, in the fourth step, the intermediate was cyclized via Michael addition reaction between the sulfur of the benzimidazole ring and the α,β-unsaturated carbonyl bond to produce a tricyclic benzimidazole–thiazinone derivative as the final product.

**Figure 2:**
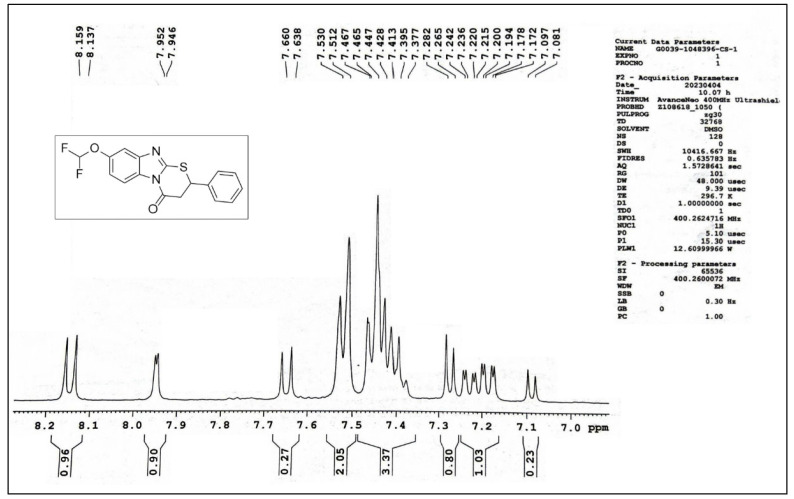
^1^H NMR spectrum of compound **CS1,** representing the aromatic region.

**Figure 3:**
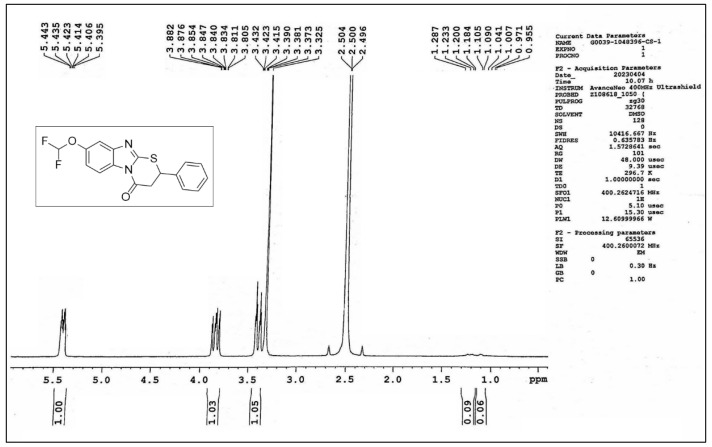
^1^H NMR spectrum of compound **CS1,** representing the aliphatic region.

The structures of newly synthesized compounds were confirmed utilizing various spectroscopic techniques, such as FT-IR, 1D NMR (^1^H, ^13^C, DEPT experiments), 2D NMR (HMQC), and mass spectrometry. All the data were found to accord with the proposed structure. In the IR spectra of new benzimidazole–thiazinone derivatives, strong absorption peaks due to stretching vibrations appeared at 3048–3084 cm^−1^ (*sp*^2^ C-H), 2903–2970 cm^−1^ (*sp*^3^ C-H) and 1678–1718 cm^−1^ (C = O). The ^1^H NMR spectra of all of the synthesized compounds exhibited signals for the protons of the vicinal CH_2_ (two multiplet in the range of 3.19–3.88 ppm) and CH (multiplet around 5.34–5.45 ppm) groups. The signals with aromatic protons appeared in the expected region (6.5–8.0 ppm). In ^13^C NMR of these compounds, characteristic signals for carbonyl carbon appeared at 167.1–172.5 ppm and for aliphatic carbon (CH and CH_2_) in the 41.00–43.00 ppm range. Further, a full assignment of the ^1^H NMR peaks for compound **CS1** is provided in Figure 2 and Figure 3. In Figure 2, a doublet peak at 8.15 ppm with a *J* value of 8.8 Hz might belong to the proton of the benzimidazole ring which was *ortho* coupled with an adjacent proton. The doublet peak at 7.95 ppm with a *J* value 2.4 Hz was also found to correspond with the proton of the benzimidazole ring, demonstrating *meta* coupling. Furthermore, the presence of a doublet signal at 7.53 with a coupling constant of 7.2 Hz might be related to two *ortho* protons of the benzene ring. A multiplet peak, appearing at 7.46–7.37 ppm, was also found to correspond with three protons of the benzene ring at *meta* and *para* positions. Another signal with a doublet (*J* = 6.8 Hz) appeared at 7.28 ppm for one proton of the benzimidazole ring, followed by a set of doublets with a small coupling constant in a unique environment, possibly corresponding with the proton of the difluorometoxy (CHF_2_) group at 7.22–7.17 ppm *δ* value. Further, Figure 3 shows the proton peaks in the aliphatic region. A peak, appearing at 5.44 ppm in multiplet, was found to belong to the proton of the methine (CH) group. Other multiplet signals, at 3.88–3.80 ppm and 3.43–3.37 ppm, were found to correspond with the protons of the methylene (CH_2_) groups. The chemical environment of these methylene protons was slightly different and so appeared at different *δ* values. Peaks at 3.32 ppm and 2.50 ppm were found to belong to H_2_O and DMSO-*d*_6_, respectively. An additional study, Distortionless Enhancement of Polarization Transfer (DEPT)-135, confirmed the presence of secondary carbon by showing a negative peak at 41.30 ppm. (Figure S1) Further, in a 2D heteronuclear multiple quantum coherence (HMQC) NMR spectrum, the carbon–proton relationship showed that the protons at 3.82 ppm and 3.43 ppm were directly attached to the same carbon atom at 41.18 ppm and the proton at 5.42 ppm was found to be attached to the carbon at 42.80 ppm. (Figure S2) The mass spectra of all of the compounds matched with calculated values, displaying [M + H] and [M + S] peaks. All of these spectral studies have confirmed the structure of the final compounds. Further, each final compound was verified to have a purity of ≥90% before conducting any biological assays.

### 2.2. Biological Evaluation

#### 2.2.1. In Vitro Antifungal Screening of Synthesized Compounds

The antifungal efficacy of tested compounds (**CS1**–**CS10**) was studied against different *Candida* strains (*Candida albicans*, *Candida glabrata* and *Candida tropicalis*) and reported while utilizing the two methods, disc diffusions and minimum inhibitory concentrations (MIC) (Table 1 and Table 2) (Figure S3). The standard used for comparison was fluconazole (FLC). In the study, using the disc diffusion method, zones of inhibition (ZOIs), showing the inhibition of microbial growth, were observed only in the cases of **CS1**, **CS4**, **CS8** and **CS10** when using a large dose. At MIC, **CS1** gave ZOIs of diameters of 13 mm, 12.5 mm and 10.5 mm for *C. albicans*, *C. glabrata* and *C. tropicalis*, respectively, which further increased to 14 mm, 13 mm and 12 mm, respectively, at 2MIC. The diameters of the ZOIs for **CS4** were 12 mm, 13.5 mm and 11.5 mm at MIC and 13.5 mm 14.5 mm and 13 mm at 2MIC against *C. albicans*, *C. glabrata*, *C. tropicalis* respectively. Next, the diameter of the ZOIs for **CS8** were 13.5 mm, 13 mm and 12.5 mm, respectively, which further increased to 15.5 mm, 15 mm and 14 mm, respectively, at 2MIC. In the presence of **CS10**, the diameters of the ZOIs were 13.5 mm, 12 mm and 13 mm for *C. albicans, C. glabrata* and *C. tropicalis*, respectively, which further increased to 15 mm, 14.5 mm and 14 mm, respectively, at 2MIC. For fluconazole, ZOIs were found to be 21 mm at 10 μg/disc against all three *Candida* strains. The ZOIs formed in the presence of test compounds were not large in comparison with those formed by fluconazole but were clear, suggesting their fungicidal nature. Furthermore, for fluconazole, the MIC was 10 μg/mL MlC and indicated no resistance against *Candida* strains. Most of the tested compounds gave very high, more than 1000 μg/mL, MlCs and failed to show significant ZOIs. **CS1** gave MICs of 820 μg/mL for *C. albicans* and 850 μg/mL for both *C. glabrata* and *C. tropicalis*. **CS4** gave MICs of 870 μg/mL, 895 μg/mL, and 925 μg/mL and **CS8** gave MICs of 830 μg/mL, 865 μg/mL, and 890 μg/mL. Further, **CS10** gave MICs of 775 μg/mL, 820 μg/mL, and 850 μg/mL. Hence, our results show that the tested compounds, mainly **CS1**, **CS4**, **CS8** and **CS10**, showed antifungal efficacy.

#### 2.2.2. In Vitro Antibacterial Screening of Synthesized Compounds

##### Screening of the Synthesized Compounds

Utilizing the bacterial strains (*Klebsiella pneumoniae*, *Pseudomonas aeruginosa*, *Salmonella typhimurium* and *Escherichia coli*), the synthesized derivatives were examined as to whether or not they had efficacy in impeding the proliferation of bacteria. All of the compounds were screened by the following two methods: (A) disc diffusion and (B) percent inhibition with standard drug ciprofloxacin.

(A) By disc

With the disc diffusion method, none of the compounds showed a zone of inhibition against *K. pneumoniae* and *S. typhimurium*. But two compounds, **CS3** (*p*-Cl substituted) and **CS4** (*o*-OCH_3_ substituted), showed respective ZOIs of 7 mm and 8 mm against *E. coli.* This suggests that the presence of the p-Cl and o-OCH3 substituents may enhance activity against E. coli, albeit to a low extent. However, these compounds displayed better activity against *P. aeruginosa,* with ZOI values of 10 (**CS3**) and 11 mm (**CS4**), indicating a higher sensitivity of *P. aeruginosa* to these specific structural modifications. Additionally, compounds **CS2** (p-Br substituted) and **CS7** (*p*-F substituted) showed ZOIs with diameters of 8 mm and 10 mm, respectively, though only against *P. aeruginosa* (Table S1). This finding suggests that halogen substitution (Br, Cl and F) at the para position and methoxy substitution at the ortho position may contribute to enhanced efficacy against P. aeruginosa. Other compounds did not show any ZOI.

(B) By percent inhibition

Single concentration of 250 µg/mL was used for percent inhibition. The compounds exhibited selective antibacterial effects, particularly against *P. aeruginosa*. For *E. coli,* among the tested compounds, only two—**CS3** (*p*-Cl substituted) and **CS4** (*o*-OCH_3_ substituted)—showed inhibitions valued at 65 and 70 percent, respectively. However, four compounds—**CS2** (*p*-Br substituted), **CS3** (*p*-Cl substituted), **CS4** (*o*-OCH_3_ substituted) and **CS7** (*p*-F substituted)—exhibited substantial inhibition in the range of 73–88 percent against *P. aeruginosa*. (Table S2). The lower inhibition rates against E. coli compared with *P. aeruginosa* might be due to differences in cell wall structure and permeability between the two bacterial species. These findings suggest that antibacterial effectiveness may be influenced by the presence of the specific structural features of these compounds, such as halogen substitutions (e.g., Br in **CS2**, Cl in **CS3**, and F in **CS7**) and the presence of an ortho-methoxy group (-OCH_3_) in **CS4**, which might enhance their interaction with bacterial cell targets.

##### Determination of Minimum Inhibitory Concentration (MIC)

Further testing, based on their ability to inhibit bacterial growth (Table 3), focused on the potential of compounds **CS2**, **CS3**, **CS4**, and **CS7** as antimicrobials against *P. aeruginosa*. These compounds showed moderate antibacterial activity against *P. aeruginosa* that produced a minimum inhibitory concentration (MIC) of 256 µg/mL to 512 µg/mL. However, their activity against *E. coli* was lower, with an MIC of 512 µg/mL to 1024 µg/mL. Notably, in the case of halogen substituted compounds, **CS2** (*p*-Br substituted) and **CS3** (*p*-Cl substituted) exhibited no significant inhibitory effect on either bacterium, as their MIC values were very high, above 256 µg/mL. However, only **CS7** (*p*-F substituted) showed moderate inhibition of *P. aeruginosa* (MIC of 256 µg/mL). This might be because of the smaller size and higher electronegativity of the fluoro group, which might be attributed to hydrogen bond formation. However, it was not effective for *E. coli* as it gave an MIC of 1024 µg/mL. **CS8**, with fluoro substitution at the *meta* position was inactive. While **CS4** (*o*-OCH_3_ substituted) demonstrated moderate efficacy against *P. aeruginosa* (MIC of 256 µg/mL), its effect on *E. coli* was limited (MIC of 512 µg/mL). The *ortho*-methoxy substitution might influence activity by its electron-donating ability through both resonance and inductive effect. However, neither **CS5** (*m*-OCH_3_ substituted) nor **CS6** (*p*-OCH_3_ substituted) showed any inhibition. Moreover, Both **CS4** and **CS7** lacked substantial inhibitory activity (MIC > 256 µg/mL) in the case of *E. coli*. Overall, these results suggest that **CS4**, with the lowest MIC against *P. aeruginosa*, is the most promising candidate for further investigation due to its moderate and potentially selective inhibitory properties.

##### Disk Diffusion Assay

The effectiveness of **CS4** and **CS7** against bacteria was further assessed using a disk diffusion assay on agar plates (Muller Hinton Agar) at the following three concentrations: half the minimum inhibitory concentration (MIC), the MIC itself, and double the MIC. Both bacterial cultures displayed clear zones of inhibition (ZOIs) around the discs containing **CS4** and **CS7**, ranging from 6–10 mm in diameter. The specific zone sizes for each compound concentration are detailed in Table 4.

##### Combination Study

A combination study of compound **CS4** was carried forward with CIP as a standard drug against *P. aeruginosa* and *E. coli* bacterial strains to detect the synergistic antimicrobial effect. The study found that **CS4**, when combined with the antibiotic CIP, displayed a significantly enhanced antibacterial effect against both *E. coli* and *P. aeruginosa* strains. This suggests a synergistic interaction between **CS4** and CIP, meaning they work together more effectively than either compound alone (Table 5). In term of both strains, the FICI was more effective against *P. aeruginosa*, with 0.185, when compared with *E. coli*, which had a value of 0.25. This result suggests that compound **CS4** possesses a promising synergistic effect with the established antibiotic CIP. This synergy, characterized by a significant substantial drop in the minimum inhibitory concentration (MIC) of the compound, indicates its potential usefulness in combination therapy for treating strains of bacteria resistant to standard antibiotics.

ADMET analysis and physicochemical properties of synthesized derivatives.

Evaluating the in silico analyses of the physicochemical, pharmacokinetic and cytotoxicity features is crucial for generating a new pharmacophore with good potency against a specific target [28]. ADMET studies were carried out on the ligands using Schrӧdinger’s QikProp module and another computational tool, pkCSM [29], to determine the possible lead likeliness of the active compounds. For the purpose of determining these features, molecular weight (MW), hydrogen bond acceptors (HBA), number of hydrogen bond donors (HBD), number of rotatable bonds (RB), topological polar surface area (TPSA), lipophilicity (Log *P*), solubility (Log *S*), skin permeability (Log K*_p_*), and blood–brain barrier (BBB) were some of the variables [30] that had been evaluated and which are given in Table 6. The study demonstrated that all of the compounds had zero HBD and RB. The number of HBA varied slightly, mostly being around 4.5 to 5.5. TPSA values differed slightly, with the highest being 115.24 for **CS10** (*p*-NO_2_ substituted) and the lowest being 69.42 for several compounds (**CS1**, **CS2**, **CS3, CS7**, **CS8**, **CS9**). Compounds **CS4**, **CS5**, **CS6** were recorded with a TPSA value of 78.65. Compounds having lower TPSA indicated that they might have better membrane permeability and could be more easily absorbed through the gastrointestinal tract and potentially cross the blood–brain barrier. **CS10** had the highest TPSA value, suggesting that it might have lower membrane permeability. Log *P* values ranged from 3.09 (**CS10**) to 4.27 (**CS2**), indicating moderate to high lipophilicity, which could aid in membrane diffusion but may reduce aqueous solubility, as reflected by the low Log S values of all compounds (e.g., −6.28 for **CS10**), indicating low aqueous solubility. Predicted skin permeability (Log K*_p_*) was uniformly low across the data, suggesting low permeability through the skin, with **CS10** having the lowest value (−5.74) and so indicating the poorest skin penetration. Further, the BBB parameter of the QikProp module indicates that a QPlogBB value greater than 0.5 suggests good penetration into the brain, a value between −1.0 to 0.5 suggests moderate penetration, and a value less than −1.0 indicates poor penetration. Thus, based on the QPlogBB value, most of the compounds showed moderate potential for crossing the blood–brain barrier, with a QPlogBB value between 0.073–0.33, with the exception of **CS10,** which had a negative QPlogBB value (−0.884), suggesting poor BBB penetration. Overall, none of the compounds violated the Lipinski rule.

Furthermore, in silico toxicity analysis was predicted using the pkCSM platform. The predicted dataset, shown in Table 7, provided insights into the safety and potential hazards of compounds (**CS1**–**CS10**). All compounds were negative for AMES toxicity, suggesting a low mutagenic risk, except **CS10** (having an electron-withdrawing nitro group at the *para* position). As an hERG I and II inhibitor, **CS1** is the only compound that inhibits both hERG I and II channels, while the others only inhibit hERG II. hERG inhibition is crucial, as it can indicate potential cardiac toxicity. Further, the maximum tolerated dose (MTD) prediction in humans ranged from 0.037–0.102 log mg/kg/day, with **CS7** showing the lowest value (0.037 log mg/kg/day). However, **CS10** exhibited the highest value (0.102 log mg/kg/day), possibly correlating with its mutagenicity. Predicted acute toxicity (LD_50_) for rats ranged from 2.219–2.509 mol/kg, indicating a similar toxicity level across compounds, whereas chronic toxicity (LOAEL) prediction showed slightly more variation, ranging from 0.683–0.92 log mg/kg/day, reflecting differences in long-term exposure. Furthermore, predicted *T. pyriformis* toxicity, used as a proxy for environmental and aquatic toxicity, remained consistent across all compounds (0.285 log μg/L), suggesting uniform toxicity to protozoa, while predicted minnow toxicity (LC_50_) was more variable, ranging from −3.167–−1.344 log mM, with **CS10** being more toxic (−3.167 log mM). Interestingly, according to the predictive model, none of the compounds exhibit hepatotoxicity or cause skin sensitization, indicating a favorable safety profile for liver toxicity and allergic skin reactions. Overall, this analysis underscored the variability in toxicity profiles among these synthesized compounds, emphasizing the need for careful consideration of their mutagenic, cardiac, and aquatic toxicities when evaluating their safety and therapeutic use.

## 3. Materials and Methods

### 3.1. Experimental Protocol

Merck (Rahway, NJ, USA) and Aldrich Chemical Company (Milwaukee, WI, USA), Spectrochem (Mumbai, India), and BLD Pharma (Shanghai, China) were preferred for purchasing the required chemicals with a purity of around 98%. All of the reagents were of synthesis grade and no further purification was needed before use. For thin layer chromatography (TLC), precoated aluminum sheets (silica gel 60 F254, Merck, Darmstadt, Germany) were utilized, while the visualization of spots was undertaken under UV light (λ = 254 nm). The melting point of all of the synthesized derivatives was captured on an uncorrected Veego (Mumbai, India) instrument with model specification REC-22038 A2. IR spectra (in cm^−1^) were captured through an Agilent Cary 630 FT-IR spectrophotometer (Santa Clara, CA, USA). ^1^H NMR and ^13^C NMR spectra were analyzed in DMSO-*d*_6_ solvent through a Brüker (Billerica, MA, USA) Spectrospin 400 MHz and with trimethyl silane as the internal standard. The designated splitting patterns were as follow: s: singlet; d: doublet; t: triplet; m: multiplet; Ar: aromatic. The chemical shift values are given in parts per million (ppm). In ^1^H NMR chemical shifts (*δ*) for DMSO-*d*_6_ appears at *δ* 2.54, whereas in ^13^C NMR, a chemical shift for DMSO-*d*_6_ is recorded at 39.5. Furthermore, the coupling constants (*J*) are stated in hertz (Hz). Mass spectra of all of the compounds were recorded using an Agilent Quadrupole-6150, which is an LC/MS spectrometer.

#### Synthesis Protocol for the Preparation of Substituted Benzimidazole–Thiazinone Derivatives (**CS1**–**CS10**)

In a 100 mL dried round-bottomed flask, a mixture of substituted acrylic acids **C2** (1.1 mmol), using TBTU as coupling reagent (1.1 mmol), DIPEA as base (3.0 mmol) and dry DMF as a solvent, was stirred at 0 °C for 15–20 min. Then, the solution of (difluoromethoxy)-1*H*-benzo[d]imidazole-2-thiol **C1** (1.0 mmol) was added to the resulting mixture and the reaction mixture was stirred at room temperature for 36 h. The progression of reaction was tracked using TLC. When the reaction was completed, the mixture was worked up with ethyl acetate and water and then sodium bicarbonate and brine solution. Anhydrous Na_2_SO_4_ was used to dry the organic layer and evaporation of the solvent occurred under reduced pressure. Purification of synthesized derivatives was undertaken through recrystallization technique and column chromatography. The final compounds, **CS1**–**CS10**, were obtained in good to moderate yield.

*8-(difluoromethoxy)-2-phenyl-2,3-dihydro-4H-benzo[4,5]imidazo[2,1-b][1,3]thiazin-4-one* (**CS1**)

Yellow solid, yield: 72%; M.p: 131–133 °C; R*_f_* = 0.40 (EtOAc: hexane = 3:7); FT-IR (cm^−1^): 3064, 3051, 2940, 1714, 1611, 1465, 1404, 1344, 1278, 1214, 1115, 1033, 863, 812, 770, 740, 698 cm^−1^; ^1^H NMR (400 MHz, DMSO-*d*_6_) (*δ*, ppm): 8.15 (d, *J* = 8.8 Hz, 1H, Ar-H), 7.95 (d, *J* = 2.4 Hz, 1H, Ar-H), 7.53 (d, *J* = 7.2 Hz, 2H, Ar-H), 7.46–7.37 (m, 3H, Ar-H), 7.28 (d, *J* = 6.8 Hz, 1H, Ar-H), 722–7.17 (m, 1H, HCF_2_), 5.44–5.40 (m, 1H, CH), 3.87–3.81 ((m, 1H, CH_2_), 3.43–3.32 ((m, 1H, CH_2_). ^13^C NMR (100.6 MHz, DMSO-*d*_6_) (*δ*, ppm): 167.4, 153.3 (d, J = 120 Hz) 143.9, 140.5, 137.5, 132.8, 129.6 (t, *J* = 40 Hz) 127.9, 119.5, 117.7 (d, *J* = 215 Hz), 117.0, 116.1, 109.1, 43.0, 41.3. ESI-MS (*m*/*z*) calc. For C_17_H_12_F_2_N_2_O_2_S: 346.06: found: 347.11 [M + H +].

*8-(difluoromethoxy)-2-(4-bromophenyl)-2,3-dihydro-4H-benzo [4,5]imidazo [2,1-b][1,3]thiazin-4-one* (**CS2**)

Orange solid, yield: 69%; M.p: 147–149 °C; R*_f_* = 0.44 (EtOAc: hexane = 3:7); FT-IR (cm^−1^): 3084, 2970, 2692, 1718, 1618, 1473, 1416, 1351, 1278, 1115, 1021, 974, 867, 815 cm^−1^; ^1^H NMR (400 MHz, DMSO-*d*_6_) (*δ*, ppm): 8.16 (d, *J* = 8.0 Hz, 1H, Ar-H), 7.66–7.45 (m, 4H, Ar-H), 7.25–7.19 (m, 1H, HCF_2_), 7.14 (m, 1H, Ar-H), 6.96 (d, *J* = 4.0 Hz, 1H), 5.44–5.39 ((m, 1H, CH), 3.81 ((m, 1H, CH_2_), 3.46–3.37 ((m, 1H, CH_2_). ^13^C NMR (101 MHz, DMSO-*d*_6_) (*δ*, ppm): 167.1, 153.0, 143.9, 136.9, 132.5 (d, *J* = 23 Hz), 130.5 (d, *J* = 36 Hz), 122.4, 120.6, 119.6, 117.0 (d, *J* = 250 Hz), 116.2, 110.5, 109.1, 42.3, 41.0; ESI-MS (*m*/*z*) calc. For C_17_H_11_BrF_2_N_2_O_2_S: 425.25: found: 427.03 [M + H +].

*8-(difluoromethoxy)-2-(4-chlorophenyl)-2,3-dihydro-4H-benzo [4,5]imidazo [2,1-b][1,3]thiazin-4-one* (**CS3**)

Cream solid, yield: 47%; M.p: 123–125 °C; R*_f_* = 0.56 (EtOAc: hexane = 3:7), FT-IR (cm^−1^): 3059, 2903, 2652, 1713, 1628, 1590, 1436, 1360, 1278, 1098, 1033, 879, 818, 667 cm^−1^; ^1^H NMR (400 MHz, DMSO-*d*_6_) (*δ*, ppm): 8. 16 (d, *J* = 8.0 Hz, 1H, Ar-H), 7.95 (d, 1H, Ar-H), 7.66–7.26 (m, 5H, Ar-H), 7.23–7.18 (m, 1H, HCF_2_), 5.45–5.42 ((m, 1H, CH), 3.84–3.82 ((m, 1H, CH_2_), 3.46–3.41 ((m, 1H, CH_2_). ^13^C NMR (100 MHz, DMSO-*d*_6_) (*δ*, ppm): 167.4, 153.0 (d, *J* = 120 Hz), 143.9, 140.1, 136.5, 133.9, 129.9 (d, *J* = 32 Hz), 119.6, 117.7, 117.0 (d, *J* = 257 Hz), 116.1, 109.1, 106.8, 42.3, 41.2; ESI-MS (*m*/*z*) calc. For C_17_H_11_ClF_2_N_2_O_2_S: 380.02: found: 413.09 [M + S].

*8-(difluoromethoxy)-2-(2-methoxyphenyl)-2,3-dihydro-4H-benzo [4,5]imidazo [2,1-b][1,3]thiazin-4-one* (**CS4**)

Yellow solid, yield: 45%; M.p: 129–132 °C; R*_f_* = 0.47 (EtOAc: hexane = 3:7), FT-IR (cm^−1^): 3048, 2945, 2841, 2705, 1678, 1617, 1460, 1425, 1328, 1243, 1217, 1101, 1024, 928, 875, 752 cm-1; ^1^H NMR (400 MHz, DMSO-*d*_6_) (*δ*, ppm): 7.87 (d, *J* = 16 Hz, 1H, Ar-H), 7.69 (d, 1H, Ar-H), 7.43–7.39 (m, 1H, Ar-H), 7.30–7.25 (m, 1H, HCF_2_), 7.18–6.96 (m, 4H, Ar-H), 5.61–5.57 (m, 1H, CH), 3.87 (m, 1H, CH_2_), 3.84 (s, 3H, OCH_3_), 3.31–3.25 (m, 1H, CH_2_); ^13^C NMR (100 MHz, DMSO-*d*_6_) (*δ*, ppm): 172.1, 169.7 (d, *J* = 141 Hz), 158.1, 146.8, 139.1, 133.2, 130.3, 128.8 (d, *J* = 25 Hz), 122.9, 121.1, 119.7, 114.5 (t, *J* = 260 Hz), 110.5, 101.4, 56.1, 56.0, 41.7. ESI-MS (*m*/*z*) calc. For C_18_H_14_F_2_N_2_O_3_S: 376.06: found: 377.37 [M + H +].

*8-(difluoromethoxy)-2-(3-methoxyphenyl)-2,3-dihydro-4H-benzo [4,5]imidazo [2,1-b][1,3]thiazin-4-one* (**CS5**)

Peach solid, yield: 61%; M.p: 135–137 °C; R*_f_* = 0.46 (EtOAc: hexane = 3:7), FT-IR (cm^−1^): 3051, 2904, 2838, 1717, 1609, 1464, 1337, 1258, 1103, 1031, 958, 865, 783 cm^−1^; ^1^H NMR (400 MHz, DMSO-*d*_6_) (*δ*, ppm): 8.15 (d, *J* = 8.4 Hz, 1H, Ar-H), 7.94 (d, *J* = 2 Hz, 1H, Ar-H), 7.66–7.23 (m, 2H, Ar-H), 7.21–7.17 (m, 1H, HCF_2_) 7.09–6.95 (m, 3H, Ar-H), 5.39–5.35 (m, 1H, CH), 3.88–3.80 (m, 1H, CH_2_), 3.77 (s, 3H, OCH_3_), 3.42–3.32 (m, 1H, CH_2_). ^13^C NMR (100 MHz, DMSO-*d*_6_) (δ, ppm) 167.7, 160.1, 153.3, 148.9, 144.0, 139.0, 132.8, 130.8 (d, *J* = 79 Hz), 120.0, 117.1 (t, *J* = 244 Hz), 116.1, 113.8, 109.1, 55.7, 43.0, 41.4. ESI-MS (*m*/*z*) calc. For C_18_H_14_F_2_N_2_O_3_S: 376.06: found: 409.17 [M + S].

*8-(difluoromethoxy)-2-(4-methoxyphenyl)-2,3-dihydro-4H-benzo [4,5]imidazo [2,1-b][1,3]thiazin-4-one* (**CS6**)

Yellow solid, yield: 67%; M.p: 128–130 °C; R*_f_* = 0.50 (EtOAc: hexane = 3:7), FT-IR (cm^−1^): 3063, 2943, 2841, 1713, 1607, 1511, 1464, 1344, 1251, 1117, 1027, 831, 726 cm^−1^; ^1^H NMR (400 MHz, DMSO-*d*_6_) (*δ*, ppm): 8.16 (d, *J* = 7.9 Hz, 1H, Ar-H) 7.96 (d, *J* = 4.0 Hz, 1H, Ar-H), 7.65–7.26 (m, 4H, Ar-H), 7.24–7.17 (m, 1H, HCF_2_), 7.00 (d, *J =* 8 Hz, Ar-H), 5.38–5.35 (m, 1H, CH), 3.83–3.79 (m, 1H, CH_2_), 3.77 (s, 3H, OCH_3_), 3.36 (d, 1H, CH_2_); ^13^C NMR (100 MHz, DMSO-*d*_6_) (*δ*, ppm): 167.7, 159.9, 153.4 (d, *J* = 129 Hz), 148.8, 144.0, 140.5, 132.8, 130.0 (d, *J* = 76 Hz), 119.5, 117.0 (d, *J* = 211 Hz), 116.0, 109.0, 106.8, 55.6, 42.6, 41.5; ESI-MS (*m*/*z*) calc. For C_18_H_14_F_2_N_2_O_3_S: 376.06: found: 377.12 [M + H].

*8-(difluoromethoxy)-2-(4-fluorophenyl)-2,3-dihydro-4H-benzo [4,5]imidazo [2,1-b][1,3]thiazin-4-one* (**CS7**)

Light brown solid, yield: 80%; M.p: 137–140 °C; R*_f_* = 0.48 (EtOAc: hexane = 3:7), FT-IR (cm^−1^): 3076, 2932, 1713, 1603, 1509, 1466, 1341, 1232, 1145, 1118, 1031, 976, 838, 745, 696 cm^−1^; ^1^H NMR (400 MHz, DMSO-*d*_6_) (*δ*, ppm): 8.15 (d, 1H, Ar-H), 7.95 (d, 1H, Ar-H), 7.66–7.26 (m, 5H, Ar-H), 7.24–7.17 (m, 1H, HCF_2_), 5.45–5.42 ((m, 1H, CH), 3.87–3.80 ((m, 1H, CH_2_), 3.43–3.31 ((m, 1H, CH_2_). ^13^C NMR (100 MHz, DMSO-*d*_6_) (*δ*, ppm): 167.3, 153.2 (d, *J* =120 Hz), 144.0, 140.5, 133.7 (d, *J* = 90 Hz), 130.3 (d, *J* = 29 Hz), 119.6, 117.8, 117.1 (d, *J* = 257 Hz), 116.6, 116.4 (d, *J* = 39 Hz), 109.1, 106.9, 42.3, 41.3; ESI-MS (*m*/*z*) caled. For C_17_H_11_F_3_N_2_O_2_S: 364.05: found: 397.15 [M + S].

*8-(difluoromethoxy)-2-(3-fluorophenyl)-2,3-dihydro-4H-benzo [4,5]imidazo [2,1-b][1,3]thiazin-4-one* (**CS8**)

Cream solid, yield: 68%; M.p: 143–145 °C; R*_f_* = 0.47 (EtOAc: hexane = 3:7), FT-IR (cm^−1^): 3075, 2948, 1713, 1618, 1591, 1468, 1342, 1257, 1137, 1032, 958, 877, 789, 689 cm^−1^; ^1^H NMR (400 MHz, DMSO-*d*_6_) (*δ*, ppm): 8.15 (d, 1H, Ar-H), 7.66–7.25 (m, 6H, Ar-H), 7.24–7.17 (m, 1H, HCF_2_), 5.43 ((t, *J* = 8.4 Hz, 1H, CH), 3.89–3.82 (m, 1H, CH_2_), 3.47–3.33 ((m, 1H, CH_2_). ^13^C NMR (100 MHz, DMSO-*d*_6_) (*δ*, ppm): 172.2, 158.1 (d, *J* = 120 Hz), 153.6, 148.7, 145.3, 142.2, 138.0 (d, *J* = 39 Hz), 134.4 (t, *J* = 32 Hz), 132.8, 122.5, 121.8 (t, *J* = 251 Hz), 121.0, 113.9, 47.8, 46.1. ESI-MS (*m*/*z*) caled. For C_17_H_11_F_3_N_2_O_2_S: 364.05: found: 365.09 [M + H].

*8-(difluoromethoxy)-2-(p-tolyl)-2,3-dihydro-4H-benzo [4,5]imidazo [2,1-b][1,3]thiazin-4-one* (**CS9**)

Off-white solid, yield: 50%; M.p: 140–142 °C; R*_f_* = 0.49 (EtOAc: hexane = 3:7), FT-IR (cm^−1^): 3063, 2925, 2884, 1713, 1610, 1462, 1342, 1276, 1114, 1032, 909, 863, 818 cm^−1^; ^1^H NMR (400 MHz, DMSO-*d*_6_) (*δ*, ppm): δ 8.16 (d, *J* = 8.8 Hz, 1H, Ar-H), 7.95 (d, *J* = 2.4Hz, 1H, Ar-H), 7.66–7.24 (m, 5H, Ar-H) 7.22–7.17 (m, 1H, HCF_2_), 5.39–5.35 (m, 1H, CH), 3.86–3.79 (m, 1H, CH_2_), 3.37–3.34 (m, 1H, CH_2_), 2.31 (s, 3H, CH_3_). ^13^C NMR (101 MHz, DMSO-*d*_6_) (*δ*, ppm): 172.5, 157.0 (d, *J* = 120 Hz), 152.6, 145.3, 143.5, 139.2, 137.6, 134.9 (d, *J* = 12 Hz), 132.6, 124.3, 122.5, 121.87 (d, *J* = 256 Hz), 113.8, 47.4, 46.3, 25.9. ESI-MS (*m*/*z*) calc. For C_18_H_14_F_2_N_2_O_2_S: 360.07: found: 361.16 [M + H].

*8-(difluoromethoxy)-2-(4-nitrophenyl)-2,3-dihydro-4H-benzo [4,5]imidazo [2,1-b][1,3]thiazin-4-one* (**CS10**)

Brown solid, yield: 42%; M.p: 156–157 °C; R*_f_* = 0.53 (EtOAc: hexane = 3:7), FT-IR (cm^−1^): 3077, 2936, 2711, 1679, 1595, 1516, 1469, 1342, 1266, 1162, 1106, 1036, 850, 756, 681 cm^−1^; ^1^H NMR (400 MHz, DMSO-*d*_6_) (*δ*, ppm): 8.16 (d, *J* = 8.0 Hz, 1H, Ar-H), 7.96–7.24 (m, 6H, Ar-H) 7.23–7.17 (m, 1H, HCF_2_), 5.46–5.42 (m, 1H, CH), 3.84–3.81 (m, 1H, CH_2_), 3.44–3.37 (m, 1H, CH_2_). ^13^C NMR (100 MHz, DMSO-*d*_6_) (*δ*, ppm): 169.7, 167.2, 161.3, 153.1 (d, *J* = 119 Hz), 143.9, 140.5, 133.7, 130.2, (d, *J* = 28 Hz), 119.6, 117.0 (d, *J* = 257 Hz), 116.6 (d, *J* = 22 Hz), 109.1, 106.8, 42.2, 41.3. ESI-MS (*m*/*z*) calc. For C_17_H_11_F_2_N_3_O_4_S: 391.35: found: 392.12 [M + H].

### 3.2. Biological Evaluation

#### 3.2.1. In Vitro Antifungal Screening of Synthesized Derivatives

##### Materials and Methods

Media preparation and culture maintenance: The investigation of antifungal ability was performed against three *Candida* strains i.e., *C. albicans* ATTCC 90028, *C. glabrata* ATCC 90030 and *C. tropicalis* ATCC 750. These strains were maintained in a medical mycology lab using standard yeast extract peptone-dextrose (YPD), for which 1:2:2 ratio was taken along with 2.5% sugar medium at 4 °C. All chemicals (obtained from Merck-Bengaluru, India) used in the experiments were of analytical grade. Furthermore, the media components were bought from HiMedia (Thane, India).

##### Antifungal Susceptibility Assays

(A) Agar Disc diffusion assay

We have assessed antifungal activity by incorporating *Candida* cells (105 cell/mL) into a growth medium. Discs (4 mm) containing various concentrations of the test compounds were placed on the medium [31,32,33]. After 48 h, the diameters of any clear zones surrounding the discs (indicating inhibited fungal growth) were measured. Fluconazole (10 µg/disc) was taken as a positive control.

(B) Minimum Inhibitory Concentration

Further, we used the standardized broth dilution procedure to establish the minimum concentration for all compounds that inhibited fungal growth against the *Candida* strains. This was undertaken in accordance with the instruction provided in CLSI reference document M27-A320. This method defines the MIC as the lowest concentration that was able to minimize fungal activity by about 90% when compared with an untreated control.

#### 3.2.2. In Vitro Antibacterial Screening of Synthesized Compounds

##### Methodology

Preparation of bacterial culture: For this, we cultured various bacterial strains on agar plates at 37 °C overnight. These strains include *K. pneumoniae* ATCC 13883, *P. aeruginosa* ATCC2453, *S. typhimurium* MTCC3224, and *E. coli* MTCC443. The bacterial colonies were carefully selected from the agar plate, and were then cultured in a broth medium for testing and examination of the synthesized derivatives.

##### In Vitro Antibacterial Screening of Synthesized Compounds

(A) By disk diffusion

To evaluate the inhibitory potential of the synthesized derivatives, we examined them against a gram-positive bacterial strain *S. typhimurium* and three gram-negative bacterial strains, *E. coli*, *K. pneumoniae* and *P. aeruginosa*. In order to conduct the preliminary screening, Muller Hinton Agar (MHA) plates were utilized for the disk diffusion experiment. The disks, containing 10 mg/mL of each compound, were put onto MHA plates, and the zones of inhibition were evaluated the next day after overnight inoculation at 37 °C [34].

(B) By percent inhibition

Compounds exhibiting noteworthy inhibition zones against the examined strains proceeded to the subsequent stage, wherein the percentage of inhibition was evaluated at a 250 µg/mL concentration. For this, the protocol was followed as per [35].

##### Estimation of Minimum Inhibitory Concentration (MIC)

Utilizing the broth micro-dilution method and adhering to the recommendations established by the National Committee for Clinical Laboratory Standards (NCCLS) guidelines, MICs of the synthesized derivatives were determined [36]. After growing secondary cultures of each bacterial isolate in LB medium, the concentration was adjusted to an OD of 0.4 (about 10^8^ CFU/mL) at 600 nm, and further diluted to about 10^6^ CFU/mL. Building on initial screenings, MIC values for the chosen compounds were established against the bacterial strains, with ciprofloxacin (CIP) as a positive control. Test compounds were taken in different concentrations, ranging from 1024 μg/mL to 2 μg/mL. Then, they were added to 96-well plates that contained the contained the solutions of 100 µL nutrient broth that were used for experiment. An amount of 100 µL of a bacterial suspension (about 5 × 10^6^ cells/mL) was introduced in each well. Over the course of 24 h, the plates were shaken continuously at the speed of 120 rpm while being incubated at 37 °C. To guarantee precision, experiments were conducted in duplicate [37].

##### Disk Diffusion Assay

The disc diffusion experiment was carried out in compliance with NARMS guidelines and was conducted to assess the antibacterial qualities of various compounds, especially for those having low MIC values. The aforementioned bacterial strains were used to culture the bacterial cells overnight at 37 °C in liquid broth medium. This was followed by the inoculation of approximately 10^5^ cells/mL into molten Muller Hinton Agar (MHA) medium. Then, the solution was poured into Petri plates measuring 90 mm. Once solidified, sterilized 4 mm diameter Whatman paper disks were placed on the agar surface. Compounds at concentrations of half the MIC, the MIC, and double the MIC were put onto these discs for testing purposes. Two controls were employed in this experiment, a standard drug as the positive control, and DMSO as the negative control. The ZOIs around the disks were measured in mm [38].

##### Fractional Inhibitory Concentration Index (FICI)

By using a 96-well plate, we measured the possible synergy between a drug and ciprofloxacin (CIP). For the method used for this demonstration we followed the protocol mentioned by Rand et al. [39]. In brief, 200 µL of sterile nutritional broth were taken in 96 well plates and a dilution of ciprofloxacin was made in serial manner with concentration ranges of 8–0.0625 μg/mL across the plate. However, the serial dilution ranges for the drug were from 256–0.5 μg/mL [40]. After inoculating the plates with roughly 2 × 10^4^ freshly prepared strains, plates were incubated overnight at 37 °C. The next day, FICI was calculated using the following formula:

FICI = (MIC of drug A in combination with B)/(MIC of drug A alone) + (MIC of drug B in combination with A)/(MIC of drug B alone)

Synergy was defined by FIC indices of ≤0.5, antagonism by indices of ≥4, and results between >0.5 and <4 were considered indifferent.

## 4. Conclusions

This study successfully employed a one-pot, three-component protocol for the synthesis of novel benzimidazole–thiazinone derivatives, comprising a fused tricyclic system. This efficient method utilizes TBTU as a coupling agent and offers several advantages, including readily available starting materials, simple reaction conditions, straightforward workup procedures, and the absence of toxic catalysts. The structures of all synthesized derivatives were confirmed using various spectroscopic techniques that include LC-MS, IR, and NMR. The relation between carbon and proton was revealed by two-dimensional NMR that confirmed the formation of the desired thiazine ring. Evaluation of the antifungal and antibacterial activities of these derivatives revealed moderate antibacterial effects, particularly against *E. coli* and *P. aeruginosa*. Among all of the derivatives, the result revealed that **CS4** exhibited better antibacterial activity against a strain of *P. aeruginosa* that had a minimum inhibitory concentration (MIC) of 256 μg/mL. Furthermore, **CS4** demonstrated a synergistic effect with the established antibiotic ciprofloxacin against both *E. coli* and *P. aeruginosa* strains. These findings suggest the potential of **CS4**, particularly in combination therapy, for treating bacterial infections caused by these strains, which are becoming increasingly resistant to standard antibiotics. Future studies will focus on further optimizing the structure–activity relationship of these derivatives to enhance their potency and explore their mechanisms of action.

## Figures and Tables

**Table 1:** ZOIs of test compounds against examined fungal strains. Fluconazole (FLC) as a positive control gave a ZOI of 21 mm.

S. No.	Code	Diameter of Zone of Inhibition								
		*C. albicans* ATCC 90028			*C. glabrata* ATCC 90030			*C. tropicalis* ATCC 750		
		MIC/2	MIC	2MIC	MIC/2	MIC	2MIC	MIC/2	MIC	2MIC
1.	**CS1**	12	13	14	11	12.5	13	9	10.5	12
2.	**CS4**	11	12	13.5	11	13.5	14.5	10	11.5	13
3.	**CS8**	12	13.5	15.5	11	13	15	10	12	14
4.	**CS10**	11.3	13.5	15	10	12	14.5	12	13	14
5.	**FLC**		21			22			21	

**Table 2:** MICs of test compounds against examined fungal strains. With FLC as a positive control, the MIC was 10 µg/mL.

S. No.	Code	MIC (µg/mL)		
		*C. albicans* ATCC 90028	*C. glabrata* ATCC 90030	*C. tropicalis* ATCC 750
1.	**CS1**	820	850	850
2.	**CS2**	>1000	>1000	>1000
3.	**CS3**	>1000	>1000	>1000
4.	**CS4**	870	895	925
5.	**CS5**	>1000	>1000	>1000
6.	**CS6**	>1000	>1000	>1000
7.	**CS7**	>1000	>1000	>1000
8.	**CS8**	830	865	890
9.	**CS9**	>1000	>1000	>1000
10.	**CS10**	775	820	850
11.	**FLC**	10	12	10

**Table 3:** MIC values in µg/mL.

S. No.	Code	*E. coli*	*P. aeruginosa*
1.	**CS2**	1024	512
2.	**CS3**	512	512
3.	**CS4**	512	256
4.	**CS7**	1024	256
5.	**CIP**	0.5	0.5

**Table 4:** Disk diffusion assay of compounds **CS4, CS7** and standard drug CIP.

Compounds	Isolates	Zone of Inhibition (ZOI) in mm		
		½ MIC	MIC	2MIC
**CS4**	*E. coli*	06	07	08
	*P. aeruginosa*	07	09	10
**CS7**	*E. coli*	-	-	06
	*P. aeruginosa*	-	06	07
**CIP**	*E. coli*	18	38	42
	*P. aeruginosa*	15	31	38

**Table 5:** Determination of fractional inhibitory concentration indices of the compound with ciprofloxacin.

Bacterial Strains	MIC Alone (μg/mL)		MIC in Combination (μg/mL)		FICI	Mode of Interaction
	CS4	CIP	CS4	CIP		
*E. coli*	512	0.5	64	0.06	0.25	Synergistic
*P. aeruginosa*	256	0.5	32	0.03	0.185	Synergistic

CIP: ciprofloxacin.

**Table 6:** Predicted in silico physiochemical and pharmacokinetic properties of target compounds (**CS1–CS10**).

Code	MW	RB	HBA	HBD	TPSA	Log *P*	Log *S*	Log *K*_p_	BBB	Lipinski Violations
** CS1 **	346.06	0	4.5	0	69.42	3.65	−5.49	−5.35	0.16	0
** CS2 **	423.97	0	4.5	0	69.42	4.27	−6.21	−5.34	0.33	0
** CS3 **	380.02	0	4.5	0	69.42	4.22	−6.15	−5.11	0.32	0
** CS4 **	376.06	0	5.25	0	78.65	3.73	−5.65	−5.55	0.073	0
** CS5 **	376.06	0	5.25	0	78.65	3.73	−5.65	−5.55	0.082	0
** CS6 **	376.06	0	5.25	0	78.65	3.73	−5.65	−5.55	0.081	0
** CS7 **	364.05	0	4.5	0	69.42	3.96	−5.6	−5.38	0.269	0
** CS8 **	364.05	0	4.5	0	69.42	3.98	−5.6	−5.38	268	0
** CS9 **	360.07	0	4.5	0	69.42	4.05	−5.88	−5.17	0.14	0
** CS10 **	391.04	0	5.5	0	115.24	3.09	−6.28	−5.74	−0.884	0

**Table 7:** Predictive in silico toxicity profile of target compounds (**CS1–CS10**).

Code	AMES Toxicity	Max. Tolerated Dose (Human)	hERG I Inhibitor	hERGII Inhibitor	Oral Rat Acute Toxicity (LD_50_)	Oral Rat Chronic Toxicity (LOAEL)	Hepatotoxicity	Skin Sensitization	*T. Pyriformis* Toxicity	Minnow Toxicity (LC_50_)
** CS1 **	No	0.051	Yes	Yes	2.219	0.901	No	No	0.285	−1.712
** CS2 **	No	0.083	No	Yes	2.236	0.683	No	No	0.285	−2.489
** CS3 **	No	0.082	No	Yes	2.235	0.693	No	No	0.285	−2.343
** CS4 **	No	0.068	No	Yes	2.222	0.791	No	No	0.285	−1.465
** CS5 **	No	0.068	No	Yes	2.222	0.791	No	No	0.285	−1.465
** CS6 **	No	0.068	No	Yes	2.222	0.791	No	No	0.285	−1.465
** CS7 **	No	0.037	No	Yes	2.241	0.821	No	No	0.285	−1.796
** CS8 **	No	0.039	No	Yes	2.233	0.801	No	No	0.285	−1.344
** CS9 **	No	0.075	No	Yes	2.224	0.92	No	No	0.285	−2.126
** CS10 **	Yes	0.102	No	Yes	2.509	0.835	No	No	0.285	−3.167

## Data Availability

Data are contained within the article and Supplementary Materials.

## Supplementary Materials

The following supporting information can be downloaded at: https://www.mdpi.com/article/10.3390/antibiotics13121155/s1, Synthetic procedure of substituted benzimidazole–thiazinone derivatives (**CS1–CS10**); Figure S1: NMR (DEPT) spectrum of compound **CS1**; Figure S2: Two-dimensional NMR (HMQC) spectrum of compound **CS1**; Figure S3: antifungal activity against *Candida* species; antibacterial screening of the compounds by measuring the zones of inhibition (ZOIs) (Tables S1 and S2); copy of NMR and mass spectra: Figures S4–S13: spectral data; copy of ^1^H NMR and ^13^C NMR; Figures S14–S23: LCMS spectra of synthesized compounds. Table S1: Screening of the compounds by measuring the zones of inhibition (ZOIs) in mm. Table S2: Percent inhibition at a single concentration of 250 µg/mL.
