# Supplementary material for: A Convenient One-Pot Synthesis of Novel Benzimidazole–Thiazinone Derivatives and Their Antimicrobial Activity
Source: Antibiotics (Basel). 2024 Dec 2;13(12):1155. doi: 10.3390/antibiotics13121155 (PMC11672583; doi:10.3390/antibiotics13121155)
Supplement: Supplementary file 1 [file antibiotics-13-01155-s001.zip › antibiotics-3278063-supplementary.pdf]

Supporting Information

A convenient one-pot synthesis of novel Benzimidazole–thiazinone derivatives and their antimicrobial activity

Sabahat Samreen[1], Asghar Ali[2], Saiema Ahmedi[3], Mohammad Raghib[1], Anzarul Haque[4], Nikhat Manzoor[3], Afzal Hussain[5], Mohammad Abid[3]*, Afreen Inam[1]*

[1]*Medicinal Chemistry Laboratory, Department of Chemistry, Jamia Millia Islamia, Jamia Nagar, New Delhi 110025 India.*

[2]*Department of Biochemistry, School of Chemical and Life Sciences, Jamia Hamdard, New Delhi 110025 India.*

[3]*Department of Biosciences, Jamia Millia Islamia, Jamia Nagar, New Delhi 110025 India.*

[4]*Central Laboratories Unit, Qatar University, Doha 2713, Qatar.*

[5]*Department of Pharmacognosy, College of Pharmacy, King Saud University, Riyadh 11451, Saudi Arabia*

***Corresponding author:** *ainam@jmi.ac.in*

***Co-corresponding author:** *mabid@jmi.ac.in*

TABLE OF CONTENTS

1) **Synthetic procedure of substituted benzimidazole–thiazinone derivatives (CS1 - CS10)**
2) **DEPT and HMQC NMR spectra of compound CS1 (Figure S1–Figure S2)**
3) **Antifungal activity against *Candida* species (Figure S3)**
4) **Antibacterial screening of the compounds by measuring the zones of inhibition (ZOIs) (Table S1 and Table S2)**
5) **Copy of ^{1}H and ^{13}C NMR spectra of compounds CS1–CS10 (Figure S4–Figure S13)**
6) **Copy of LCMS spectra of compounds CS1–CS10 (Figure S14–Figure S23);**

1. Synthetic procedure of substituted benzimidazole–thiazinone derivatives (CS1–CS10)

In a 100 mL dried round-bottomed flask, a mixture of substituted acrylic acids **C2** (1.1 mmol), with TBTU as coupling reagent (1.1 mmol) and DIPEA as base (3.0 mmol) and using dry DMF as a solvent, was stirred at 0°C for 15 to 20 minutes. Then, the solution of (difluoromethoxy)-1H-benzo[d]imidazole-2-thiol **C1** (1.0 mmol) was added to the resulting mixture and the reaction mixture was stirred at room temperature for 36 hrs. The progression of the reaction was tracked using TLC. When the reaction was completed, the mixture was worked up with ethyl acetate and water and then sodium bicarbonate and brine solution. Anhydrous Na_2SO_4 was used to dry the organic layer and the evaporation of the solvent occurred under reduced pressure. Purification of synthesized derivatives was undertaken through recrystallization technique and column chromatography. The final compounds **CS1–CS10** were obtained in a good to moderate yield.

2. DEPT and HMQC NMR spectra of compound CS1

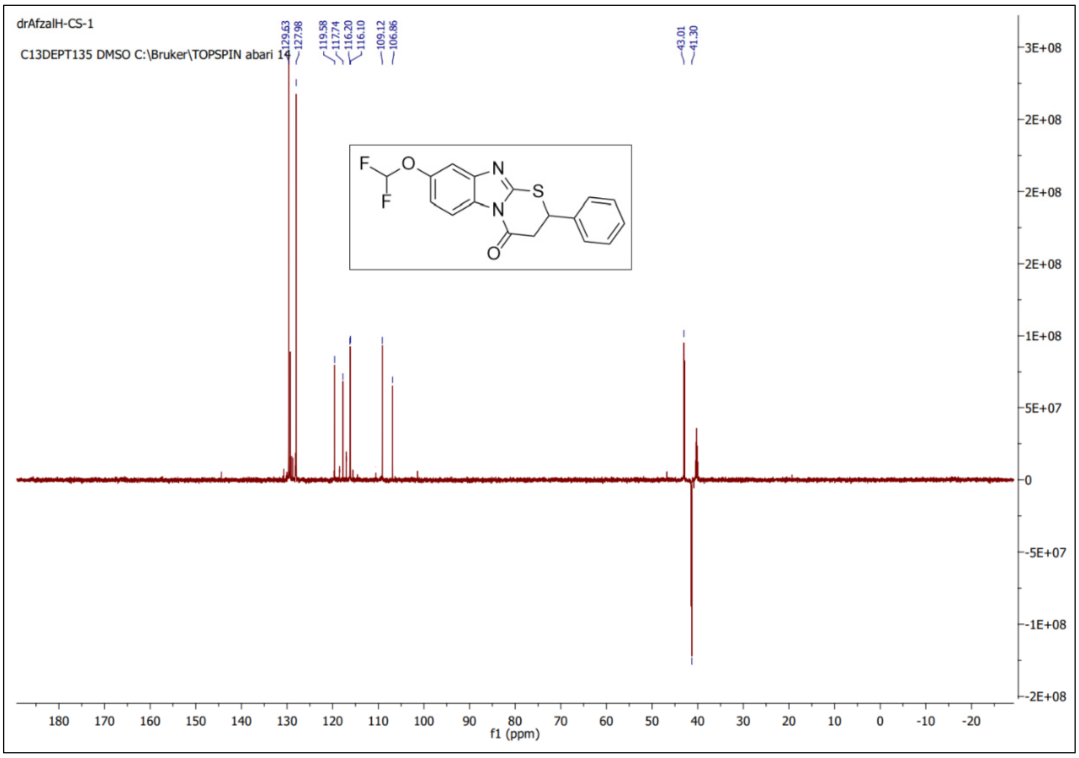

Figure S1. NMR (DEPT) spectrum of compound CS1

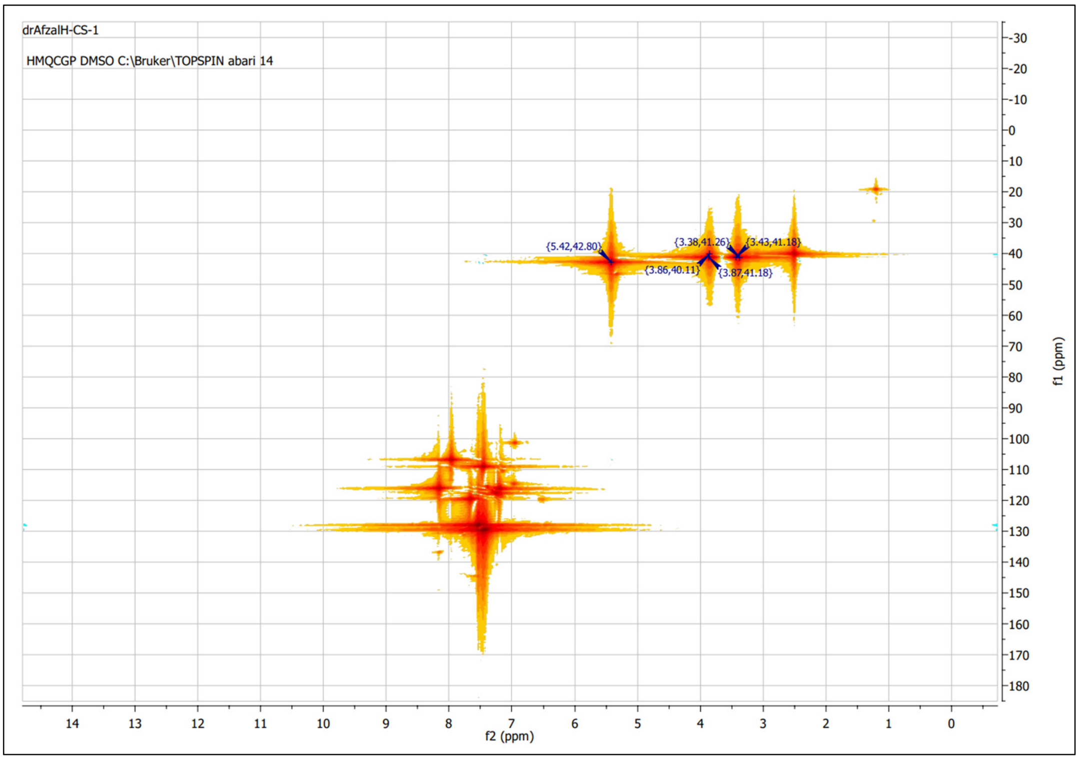

Figure S2. Two-dimensional NMR (HMQC) spectrum of compound CS1

3. Antifungal activity against *Candida* species (Figure S1)

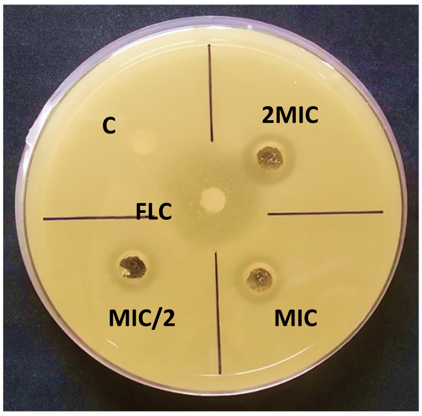

C. albicans ATCC 90028

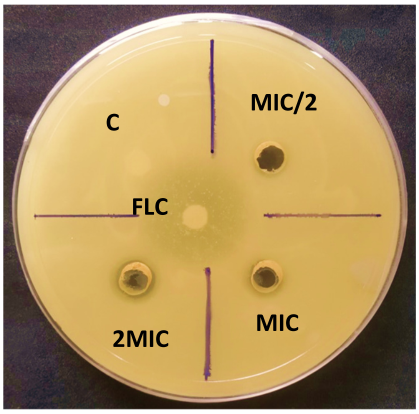

C. glabrata ATCC 90030

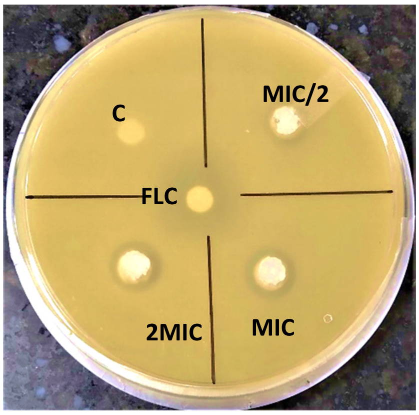

C. tropicalis ATCC 750

CS1; Activity on different *Candida* species

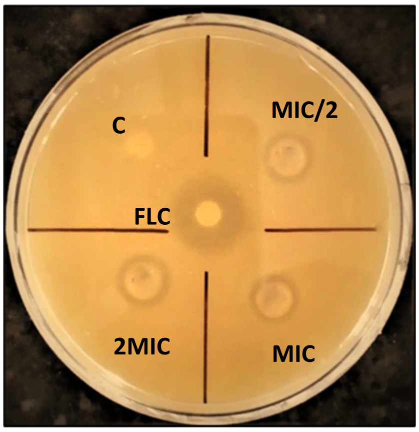

C. albicans ATCC 90028

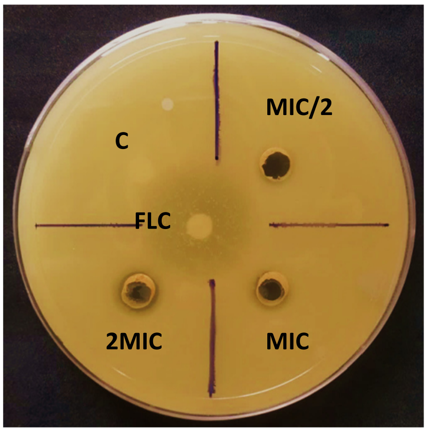

C. glabrata ATCC 90030

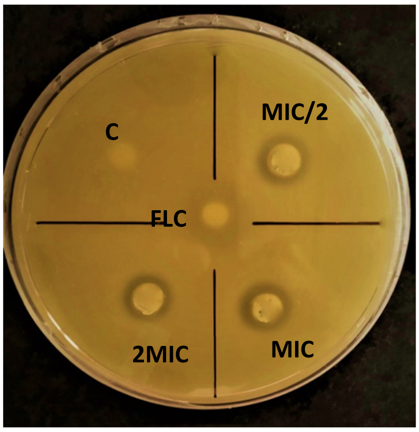

C. tropicalis ATCC 750

CS4; Activity on different *Candida* species

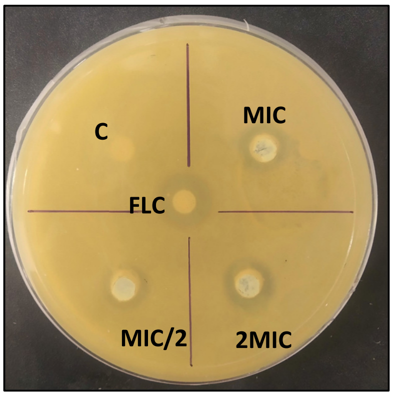

C. albicans ATCC 90028

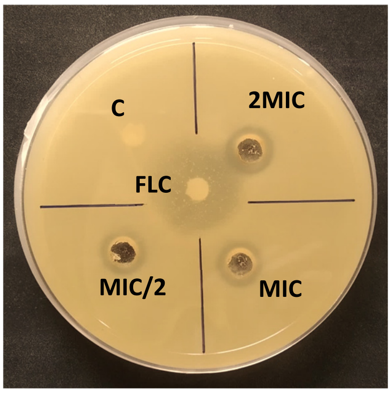

C. glabrata ATCC 90030

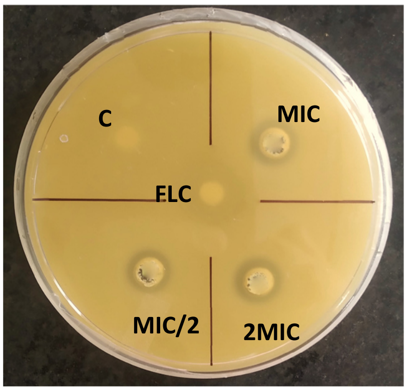

C. tropicalis ATCC 750

CS8; Activity on different *Candida* species

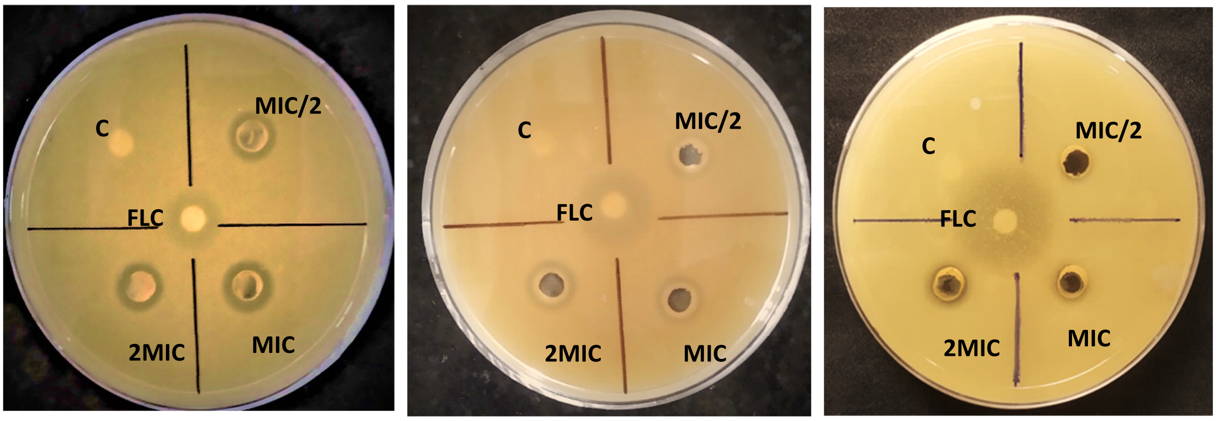

C. albicans ATCC 90028 *C. glabrata ATCC 90030* *C. tropicalis ATCC 750*

CS10; Activity on different *Candida* species

Figure S3; Antifungal activity against *Candida* species

4. Antibacterial screening of the compounds by measuring the zones of inhibition (ZOIs) (Table S1 and S2)

Table S1. Screening of the compounds by measuring the zones of inhibition (ZOIs) in mm

S. No.	Code	*E. coli*	*K. pneumoniae*	*P. aeruginosa*	*S. typhimurium*
1.	**CS1**	-	-	-	-
2.	**CS2**	-	-	08	-
3.	**CS3**	07	-	10	-
4.	**CS4**	08	-	11	-
5.	**CS5**	-	-	-	-
6.	**CS6**	-	-	-	-
7.	**CS7**	-	-	10	-
8.	**CS8**	-	-	-	-
9.	**CS9**	-	-	-	-
10.	**CS10**	-	-	-	-
11.	**DMSO**	-	-	-	-
12.	**CIP**	40	16	33	22

Table S2. Percent inhibition at a single concentration of 250 µg/mL

S. No.	Code	*E. coli*	*K. pneumoniae*	*P. aeruginosa*	*S. typhimurium*
1.	**CS1**	00	00	00	00
2.	**CS2**	05	05	73	10
3.	**CS3**	65	06	80	00

4.	**CS4**	70	11	88	07
5.	**CS5**	00	00	00	00
6.	**CS6**	05	00	03	00
7.	**CS7**	00	00	82	00
8.	**CS8**	00	02	00	00
9.	**CS9**	07	00	03	00
10.	**CS10**	00	06	00	00
11.	**DMSO**	00	00	01	00
12.	**CIP**	100	100	100	100

CIP: Ciprofloxacin

5. ^{1}H and ^{13}C NMR spectra of compounds CS1–CS10 (Figure S4–Figure S13)

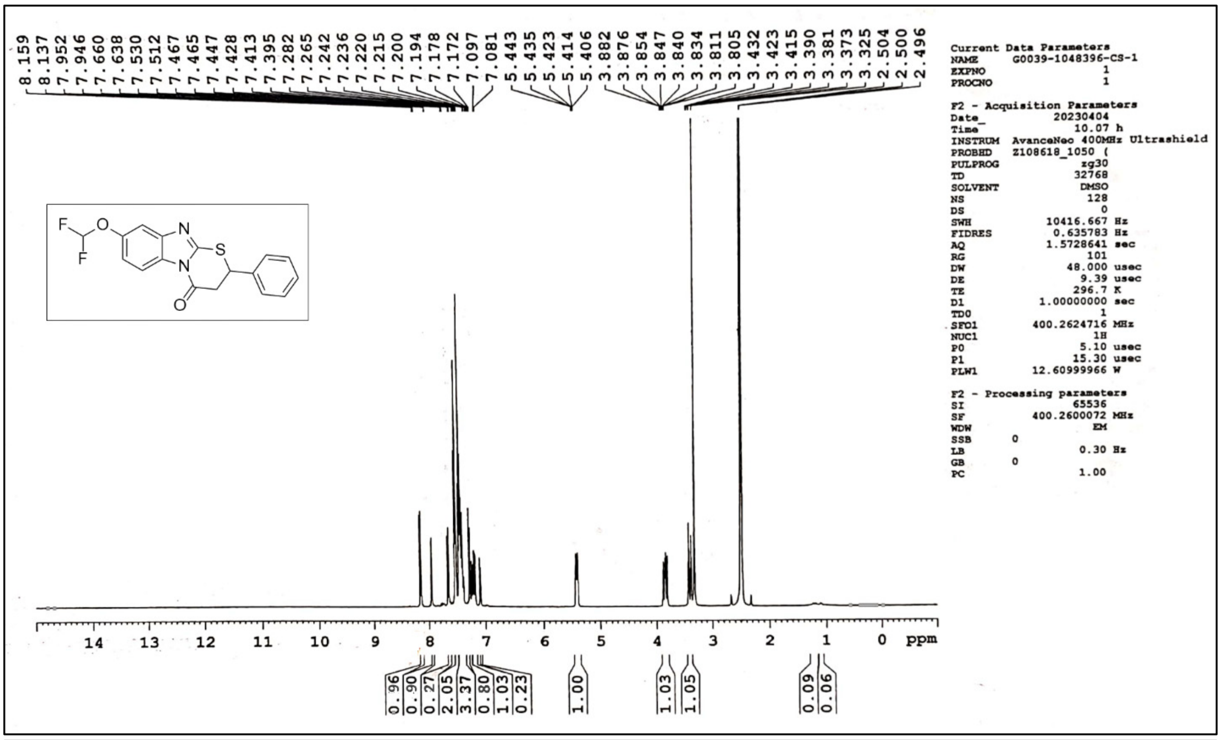

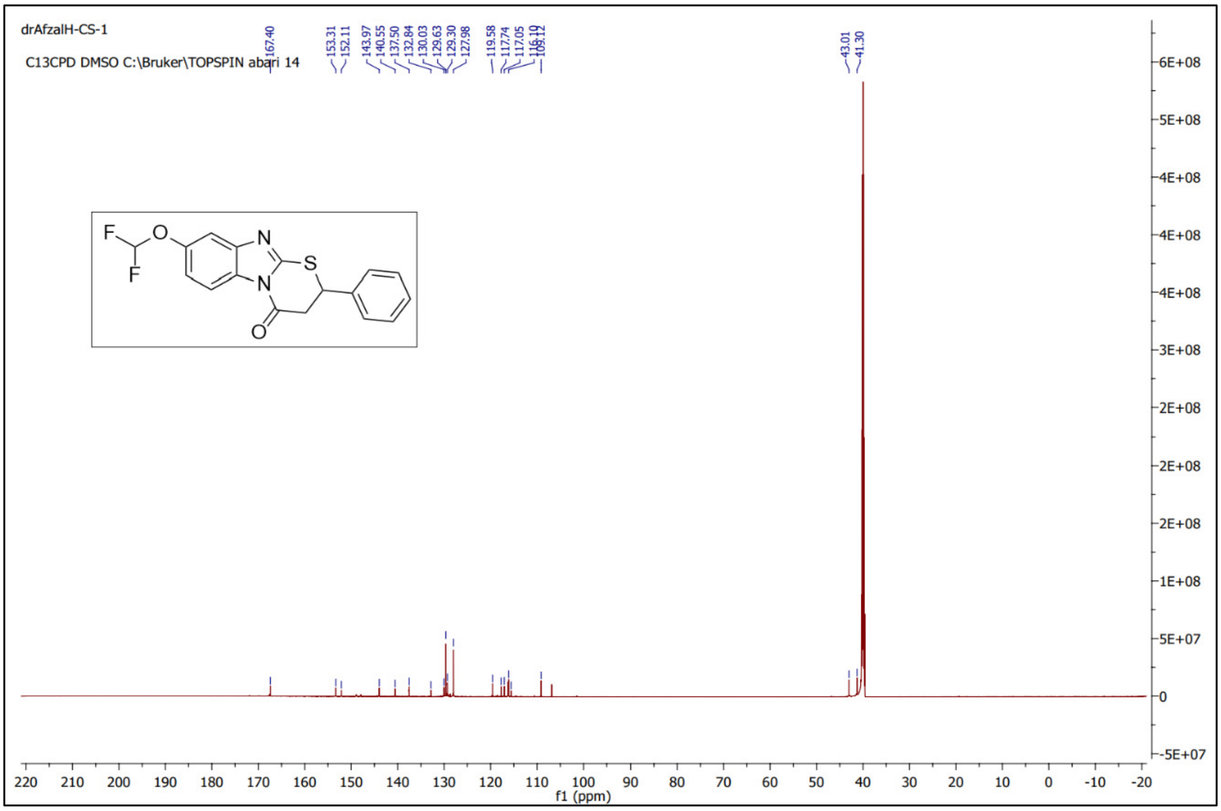

Figure S4: ^{1}H and ^{13}C NMR spectra of compound CS1

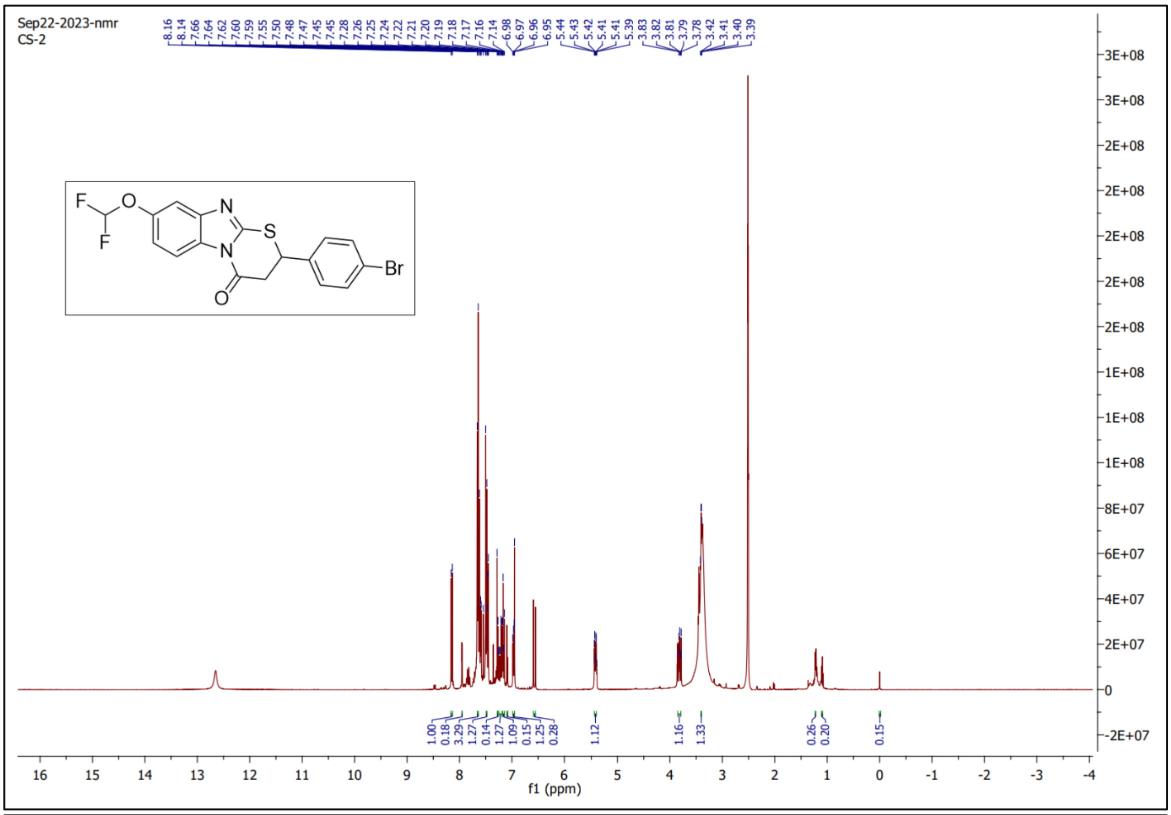

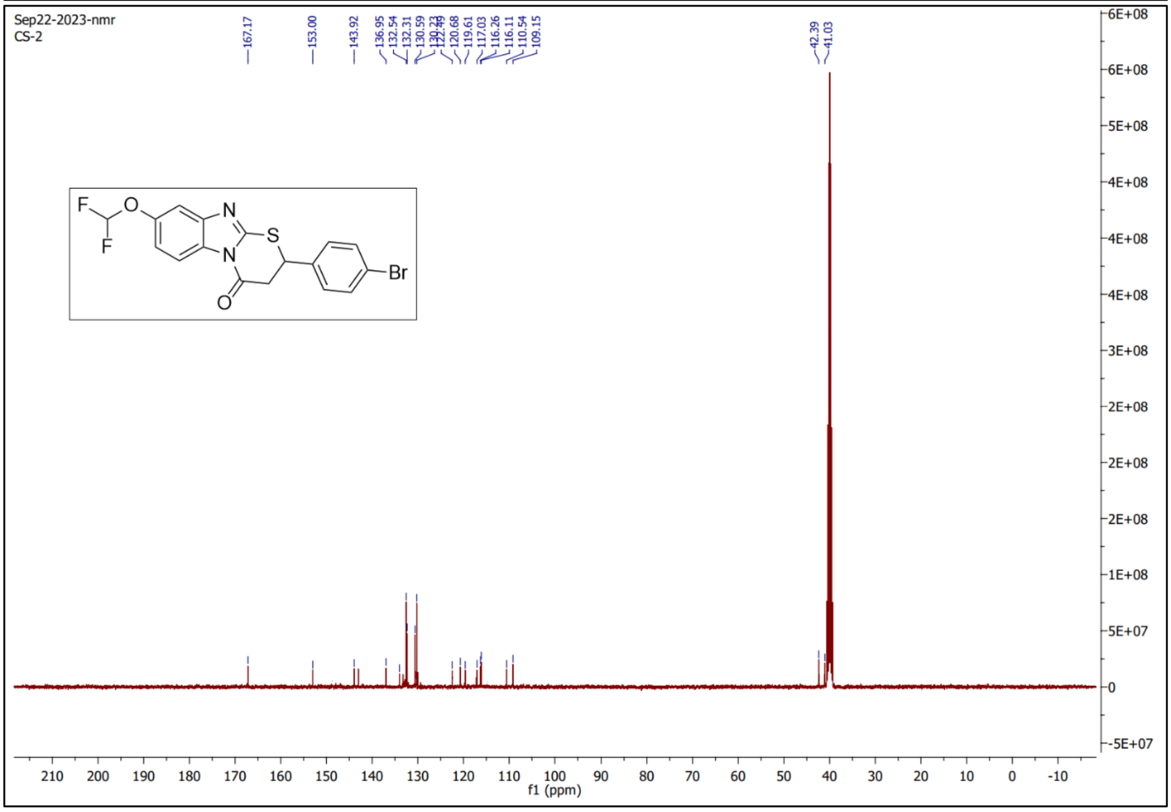

Figure S5: ^{1}H and ^{13}C NMR spectra of compound CS2

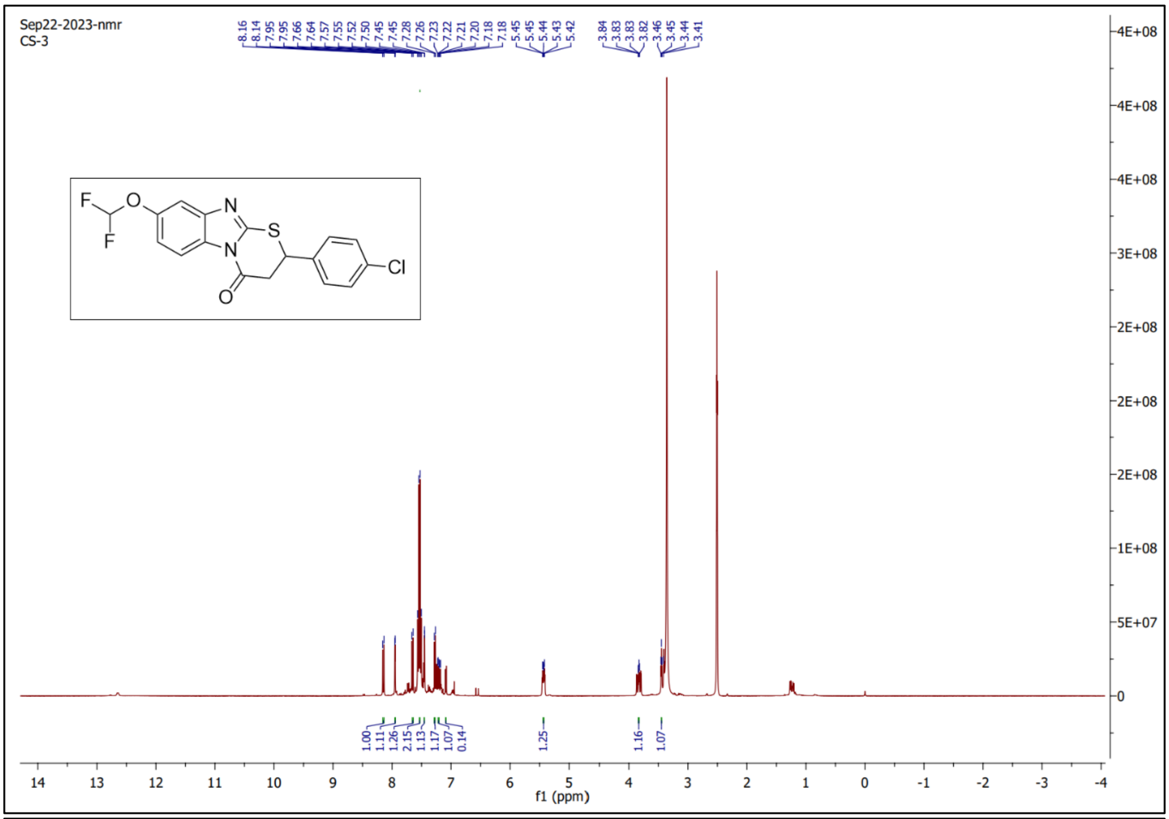

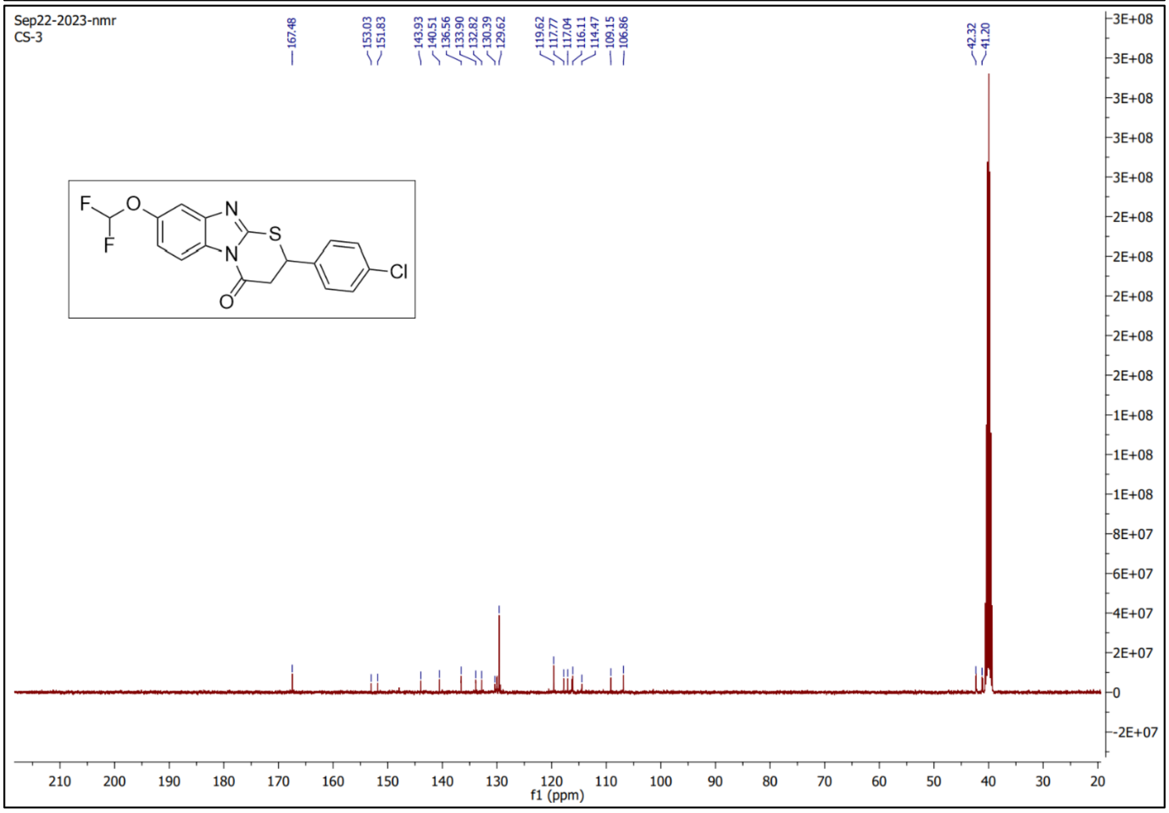

Figure S6: ^{1}H and ^{13}C NMR spectra of compound CS3

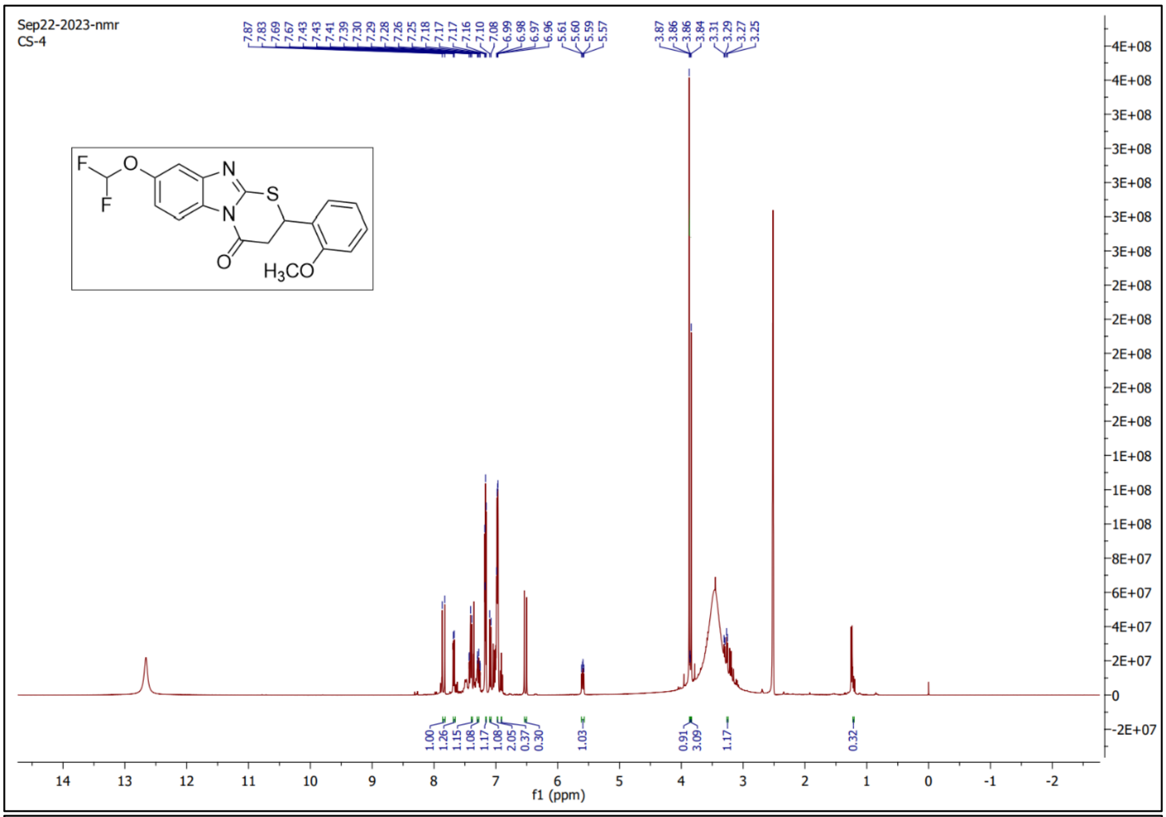

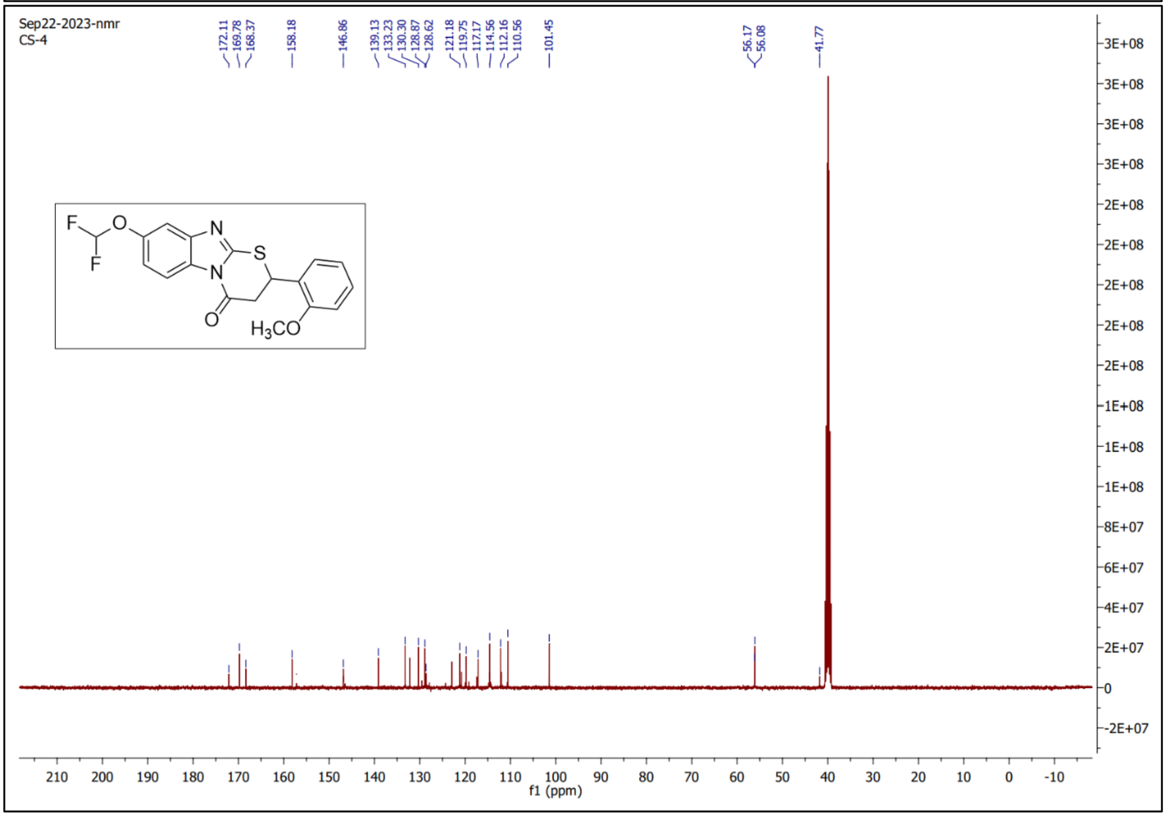

Figure S7: ^{1}H and ^{13}C NMR spectra of compound CS4

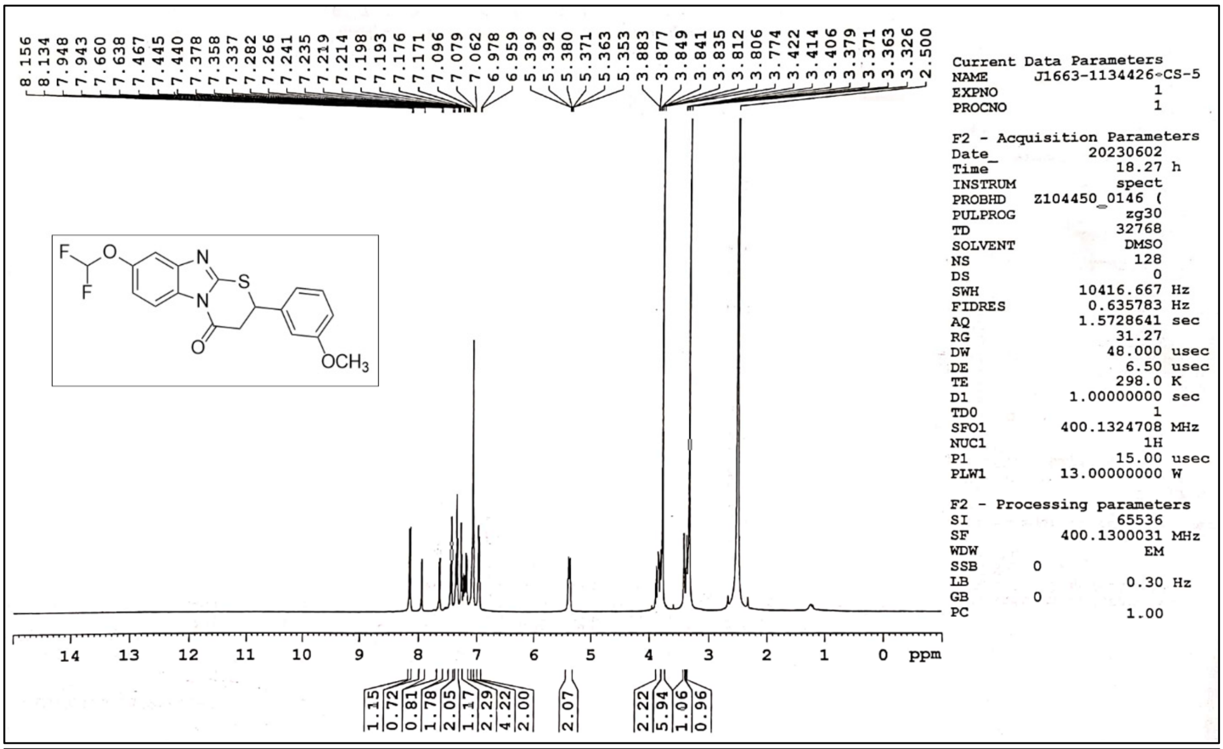

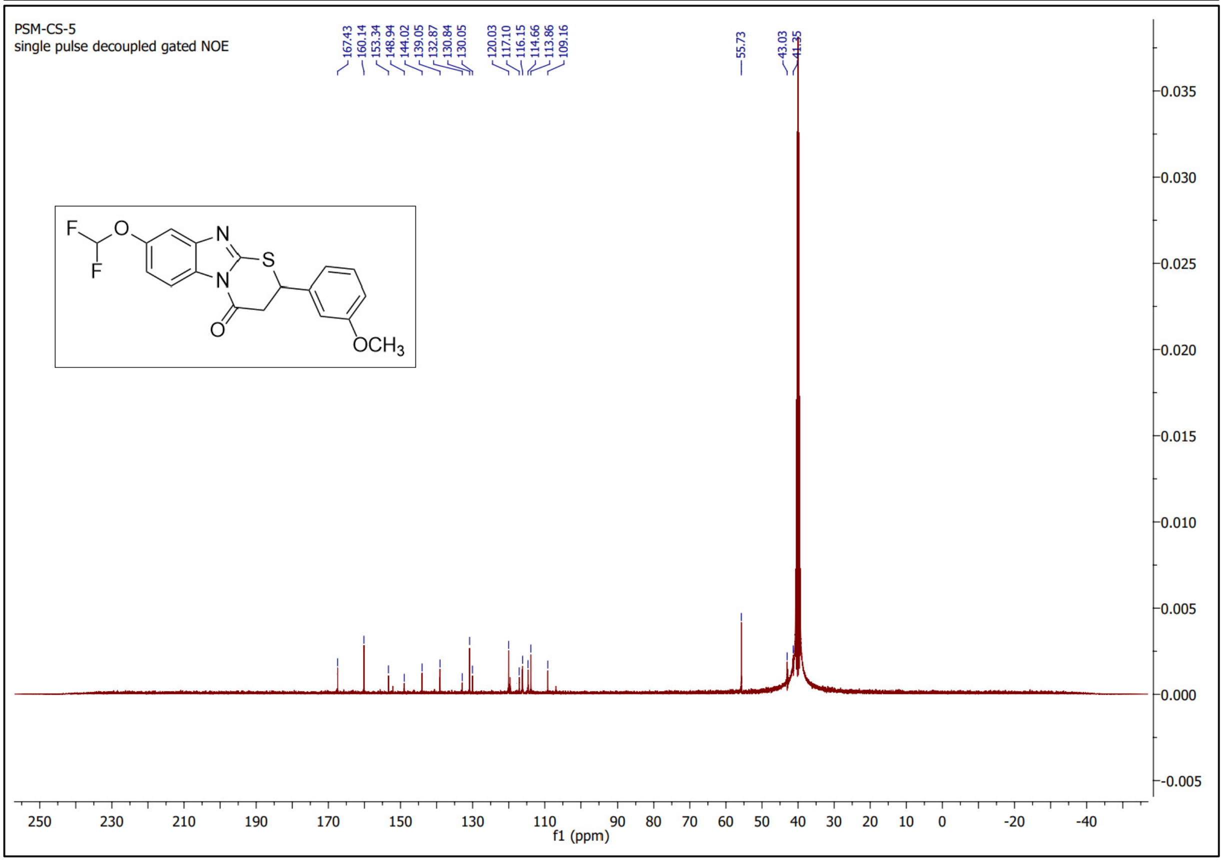

Figure S8: ^{1}H and ^{13}C NMR spectra of compound CS5

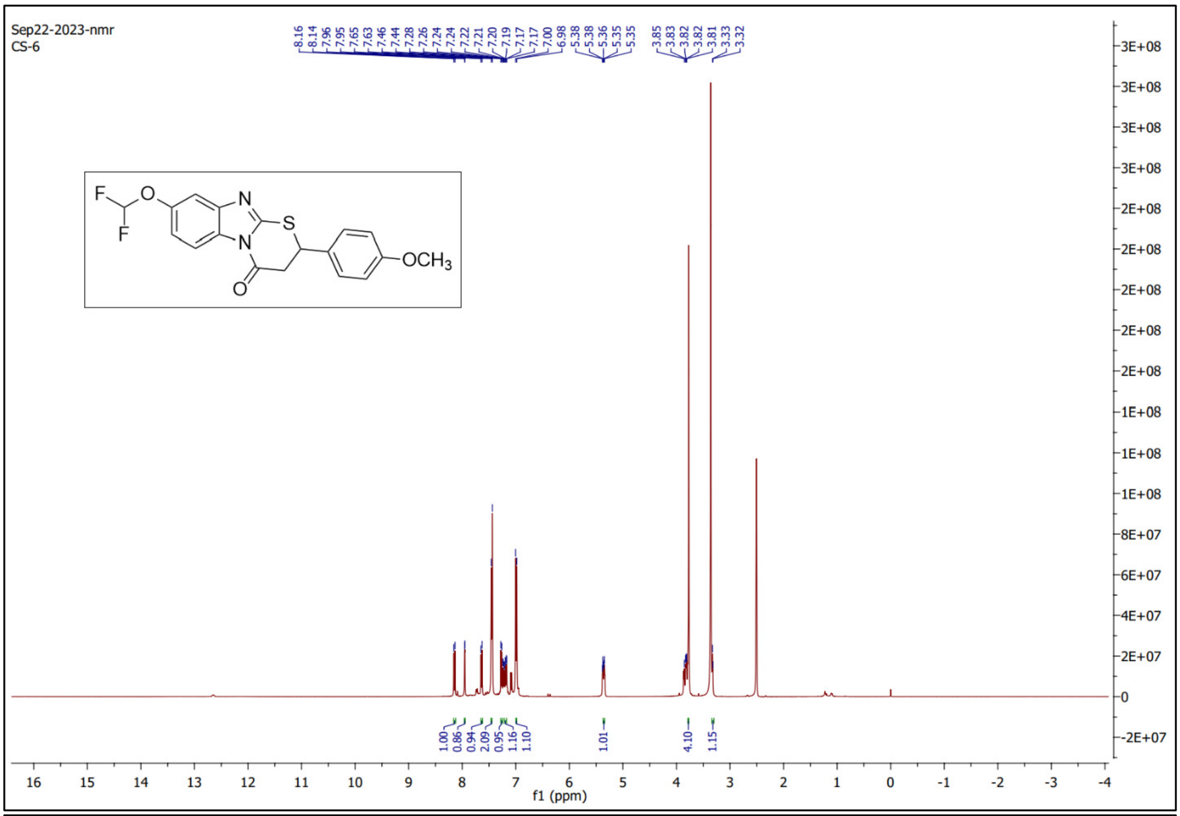

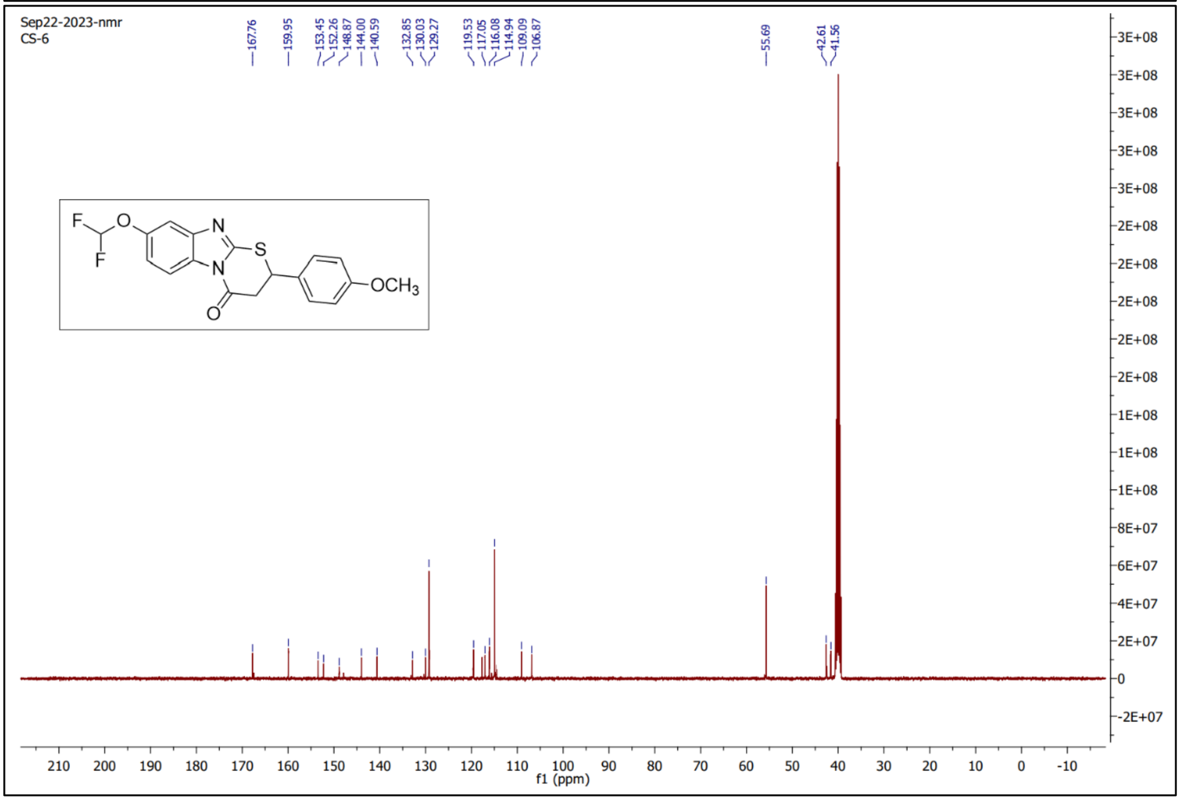

Figure S9: ^{1}H and ^{13}C NMR spectra of compound CS6

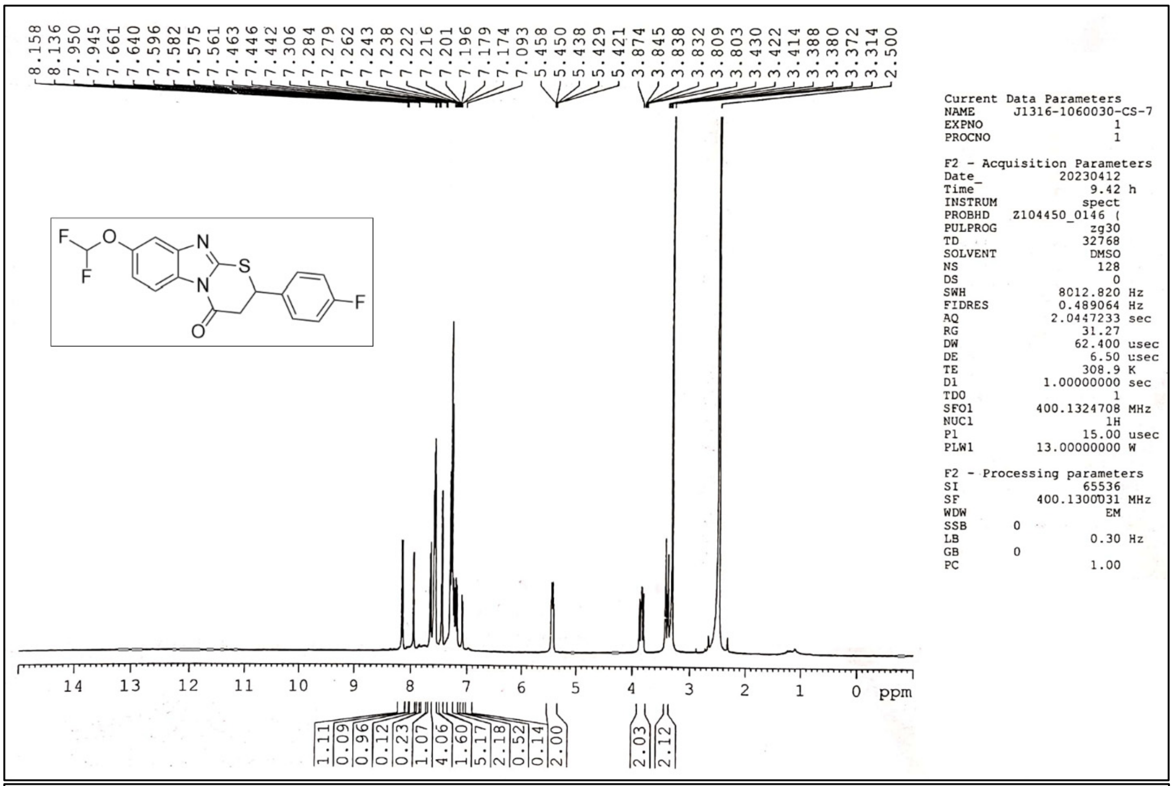

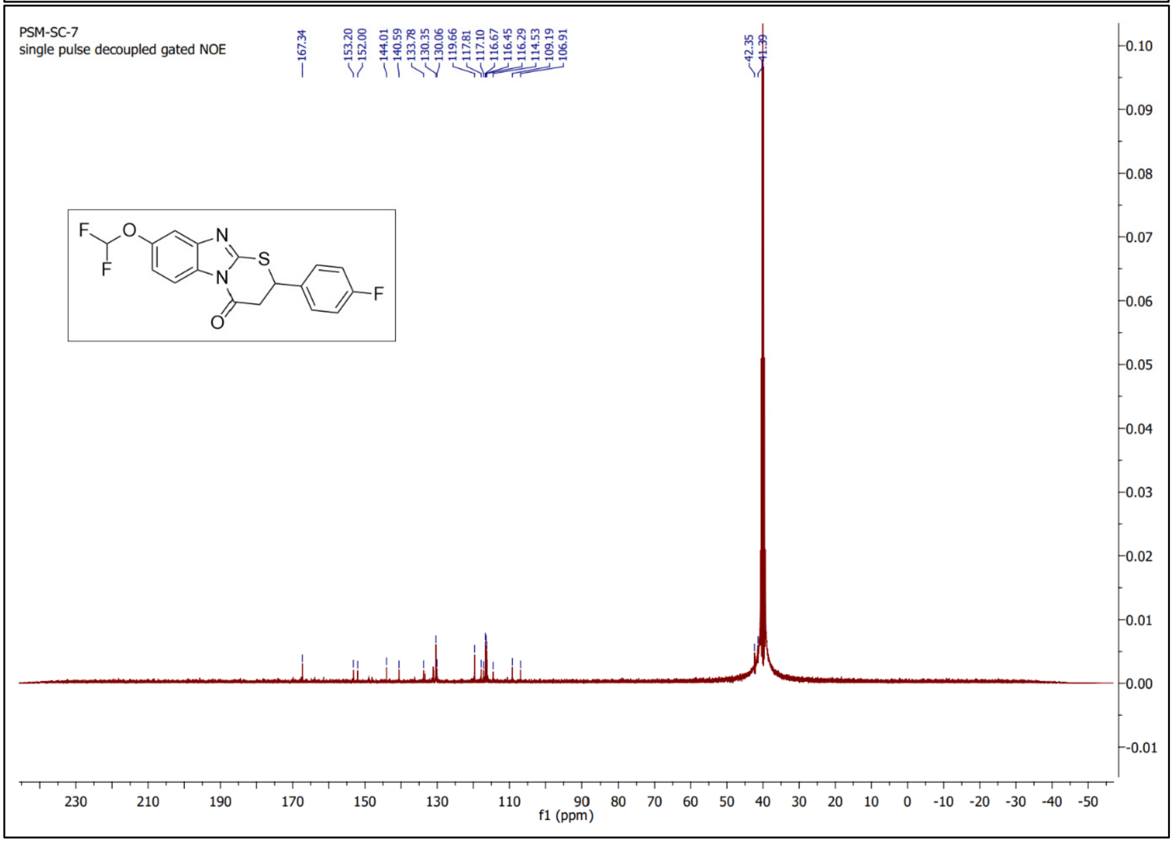

Figure S10: ^{1}H and ^{13}C NMR spectra of compound CS7

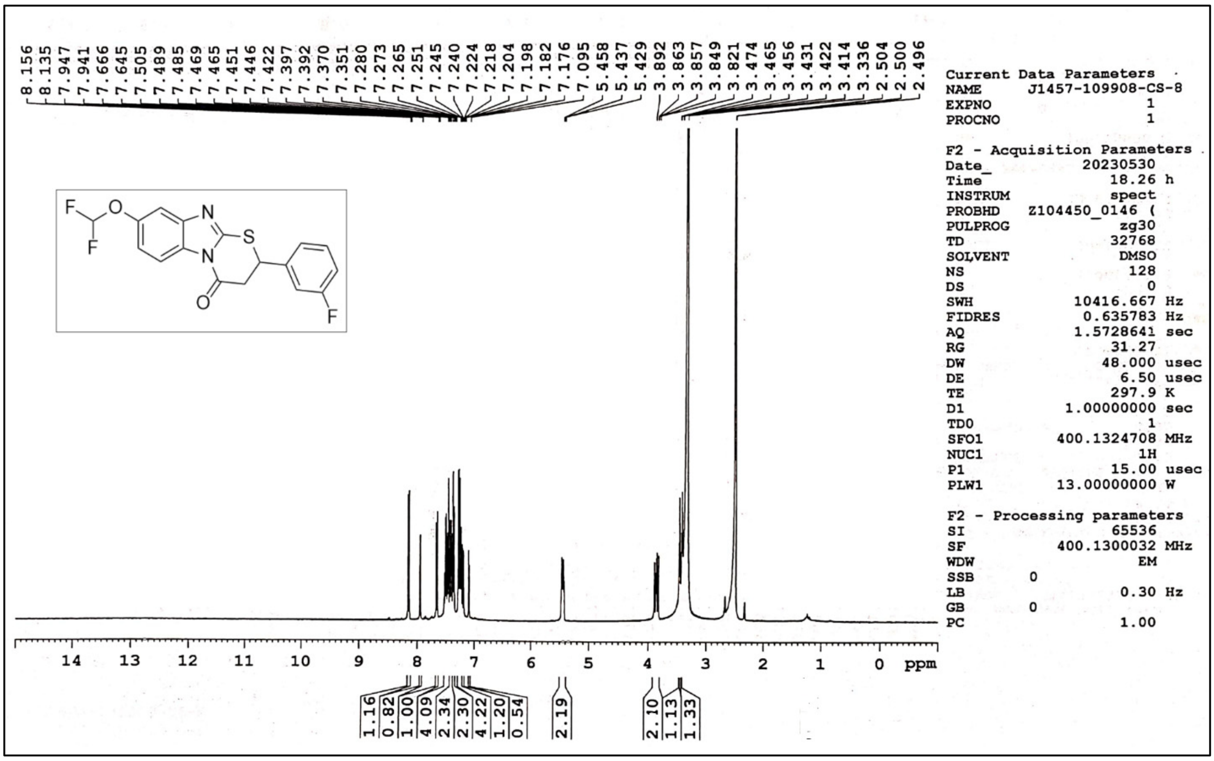

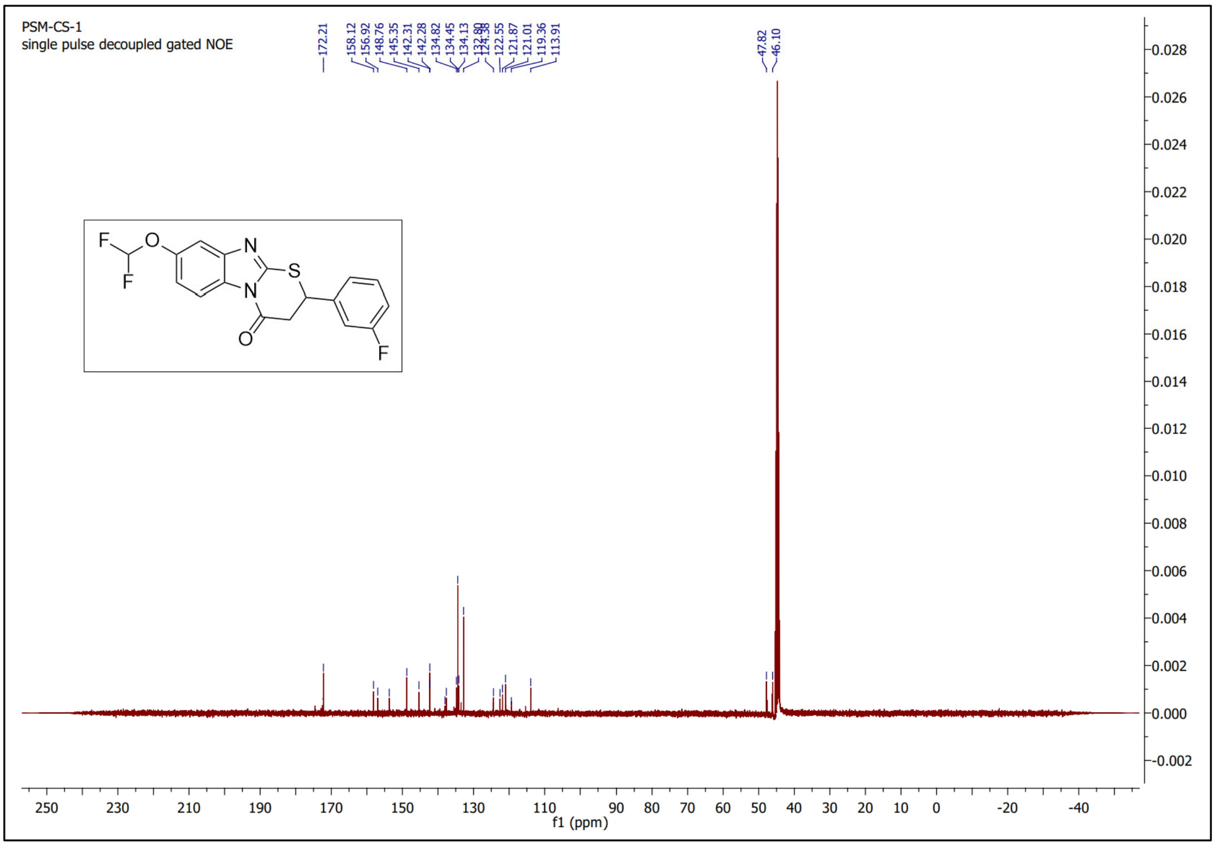

Figure S11: ^{1}H and ^{13}C NMR spectra of compound CS8

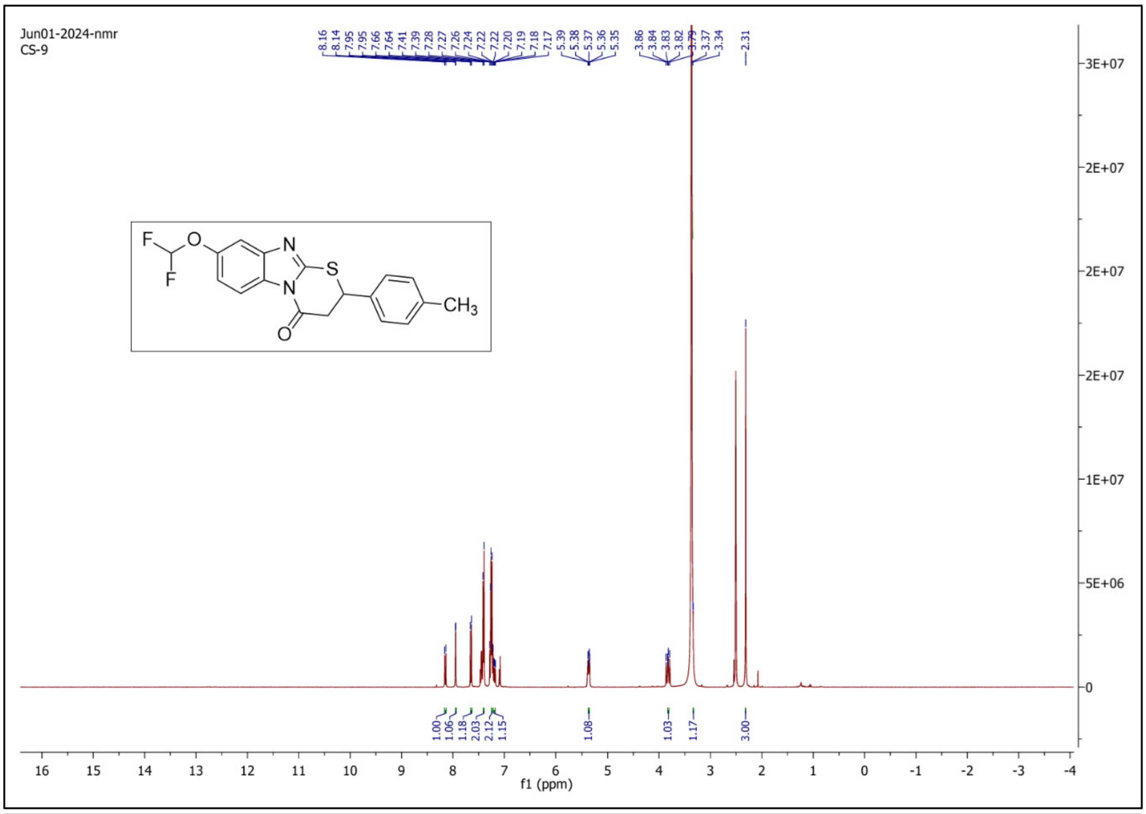

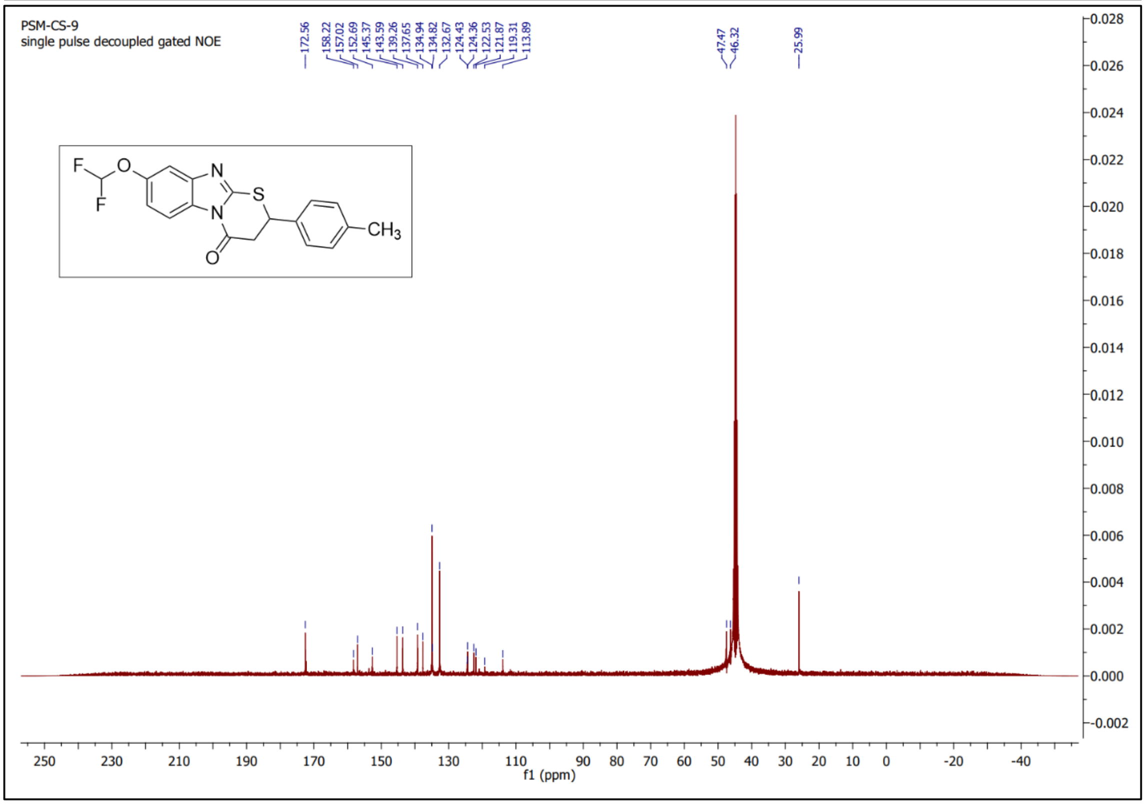

Figure S12: ^{1}H and ^{13}C NMR spectra of compound CS9

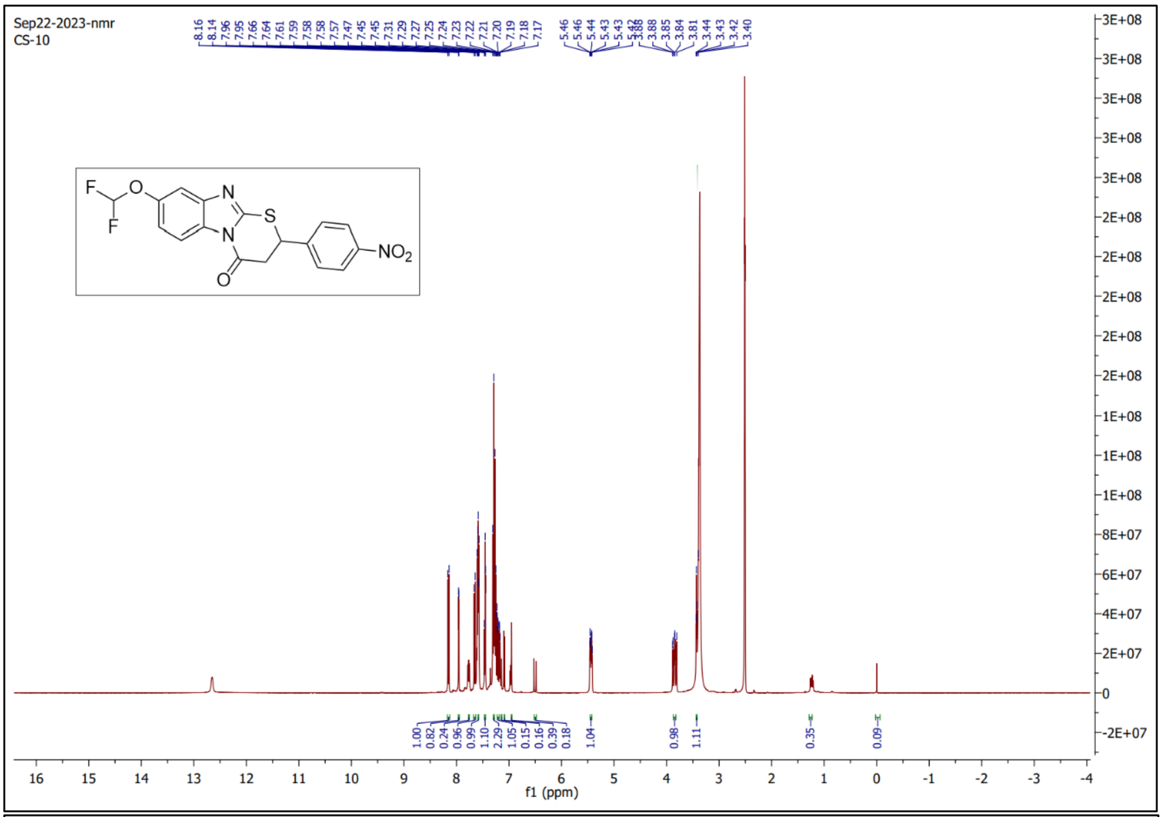

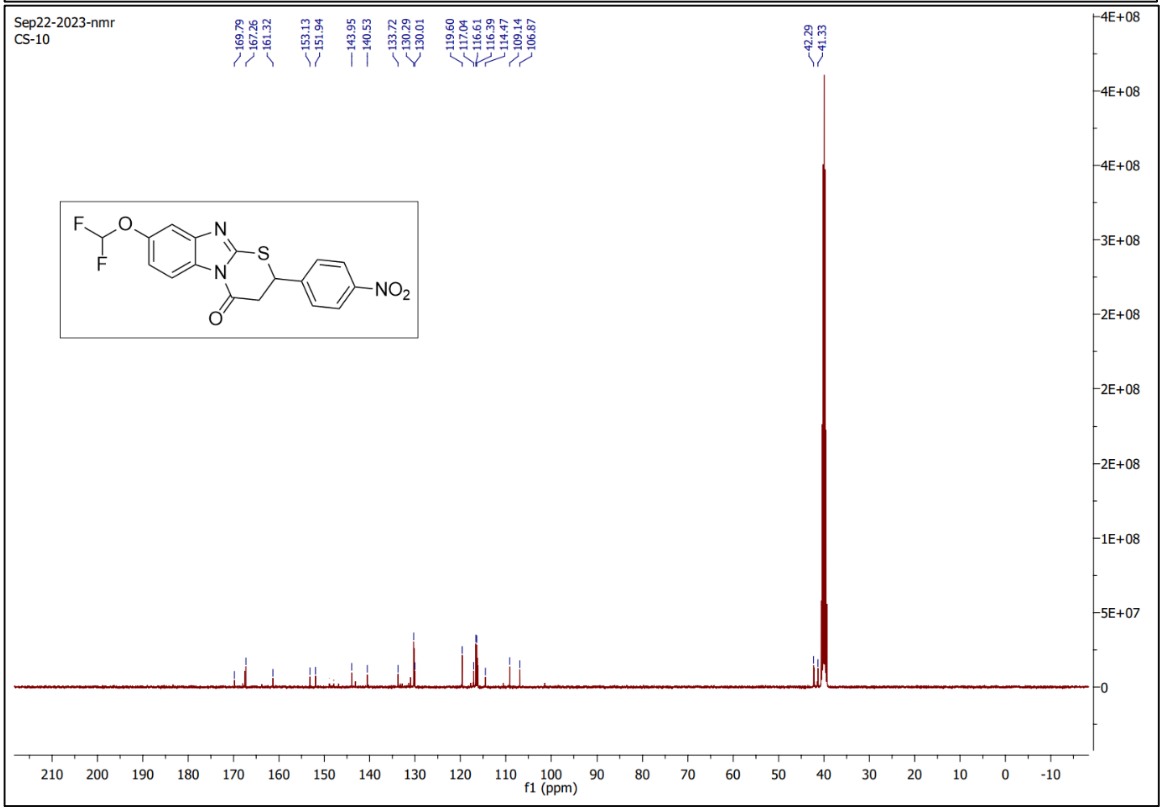

Figure S13: ^{1}H and ^{13}C NMR spectra of compound CS10

6. Copy of LCMS spectra of compounds CS1 -CS10 (Figure S14 -Figure S23);

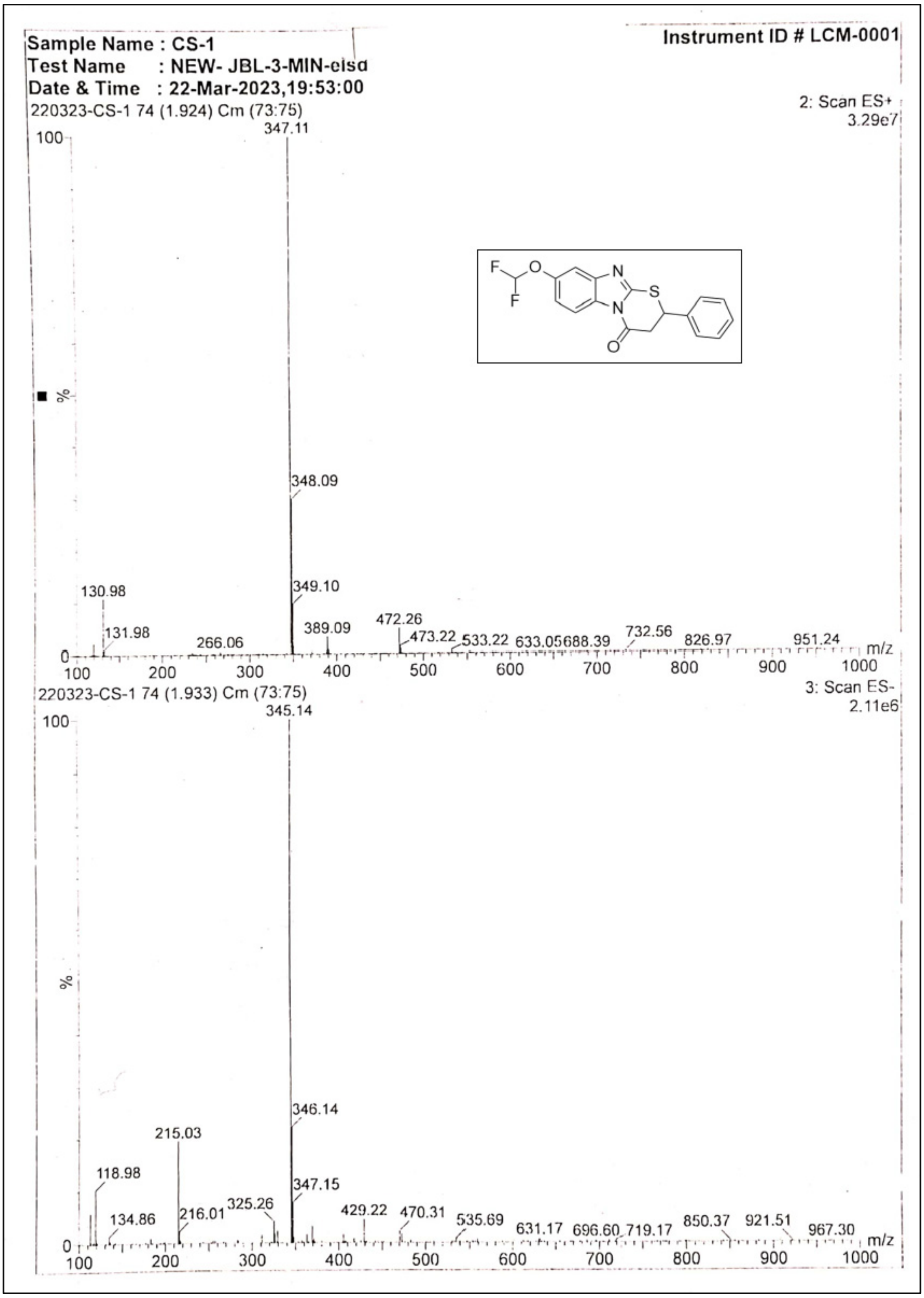

Figure S14: Compound CS1

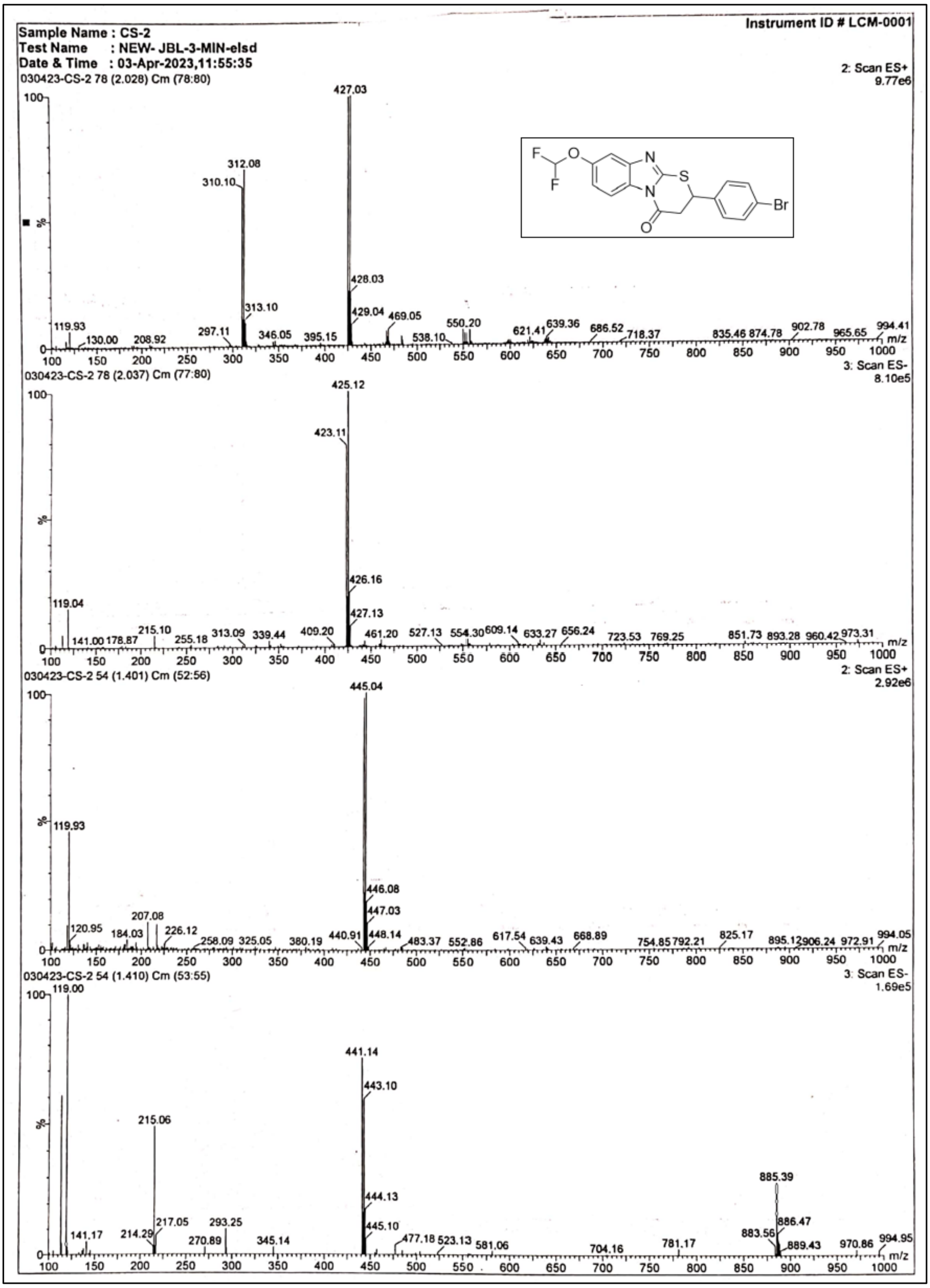

Figure S15: Compound CS2

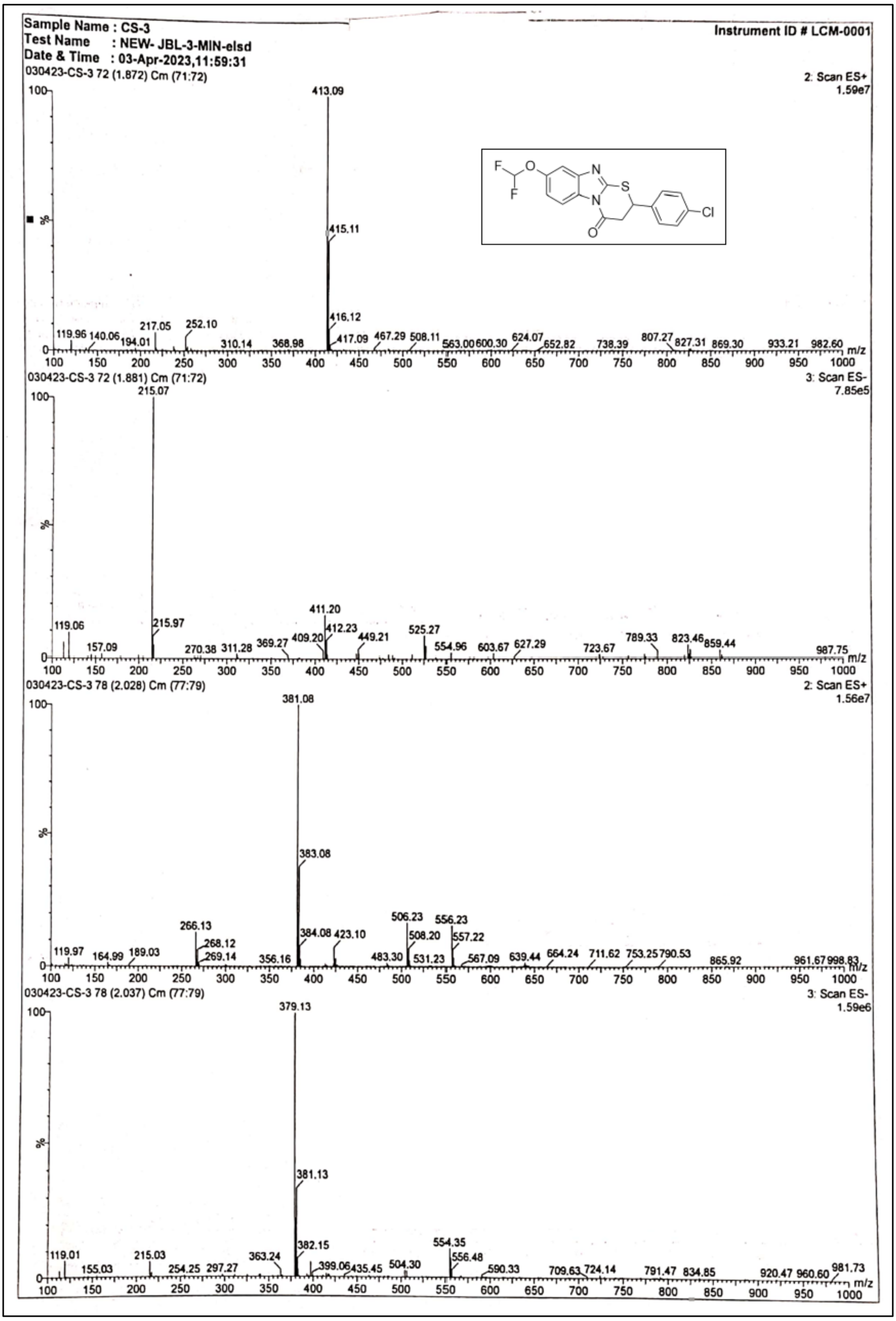

Figure S16: Compound CS3

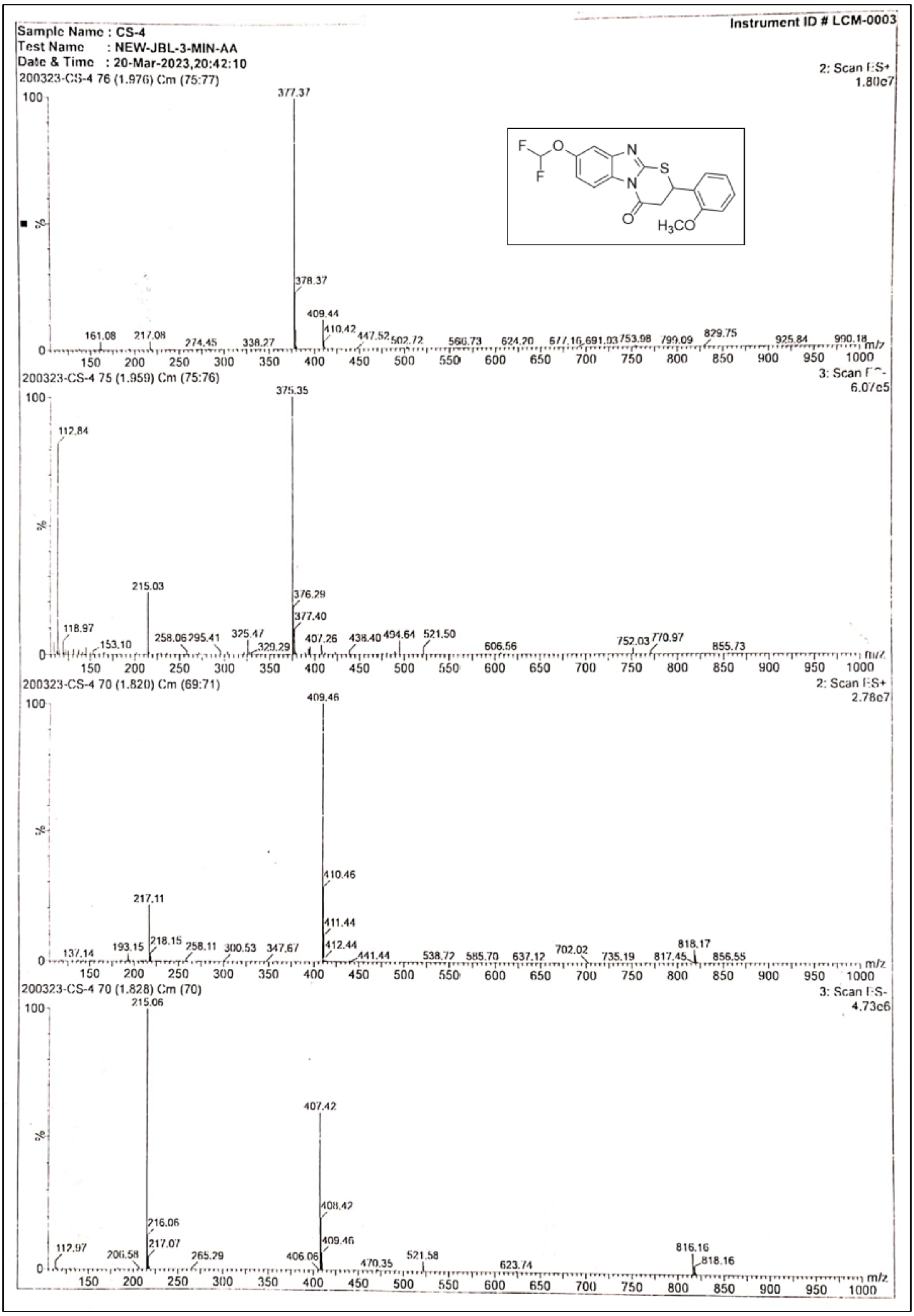

Figure S17: Compound CS4

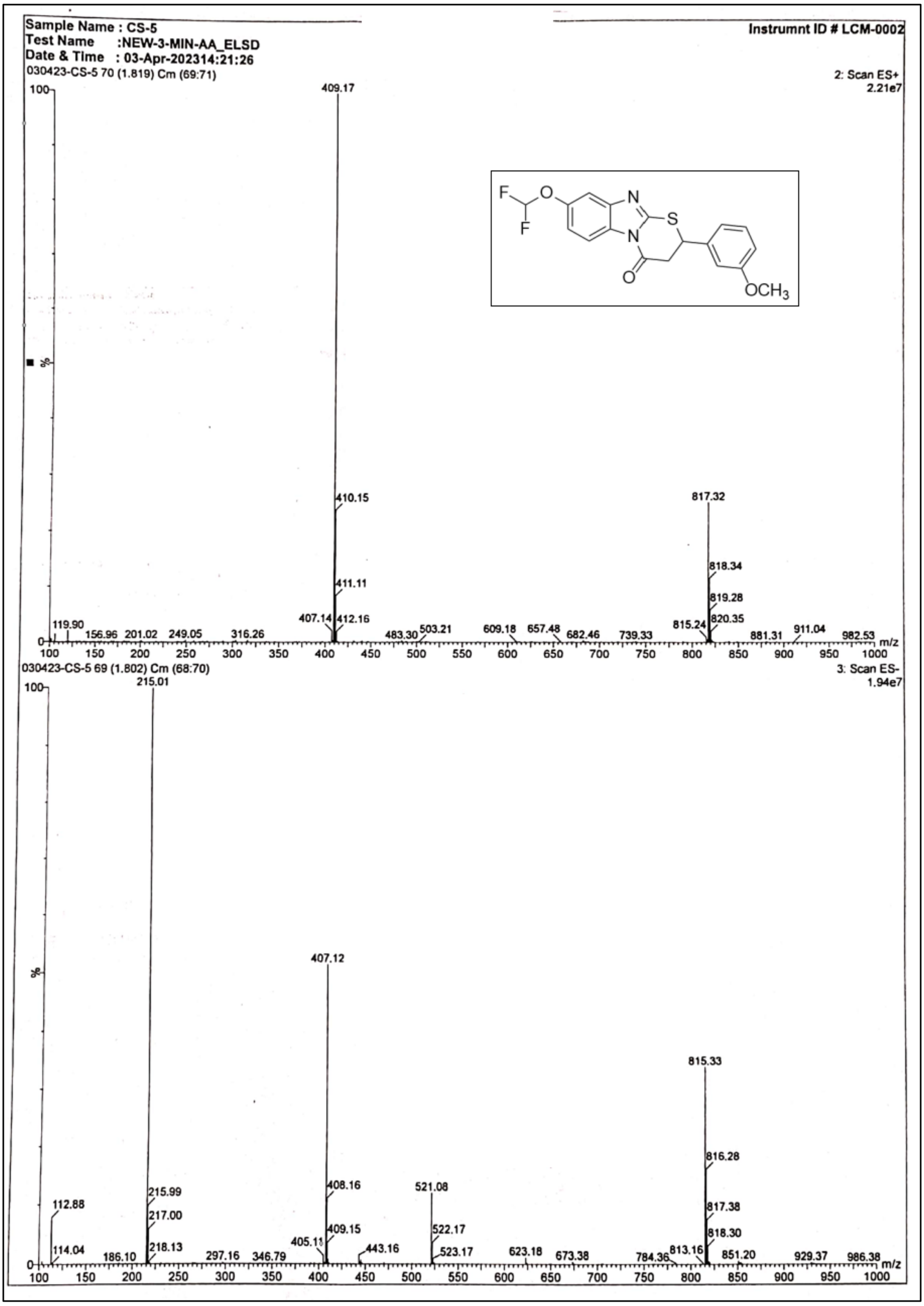

Figure S18: Compound CS5

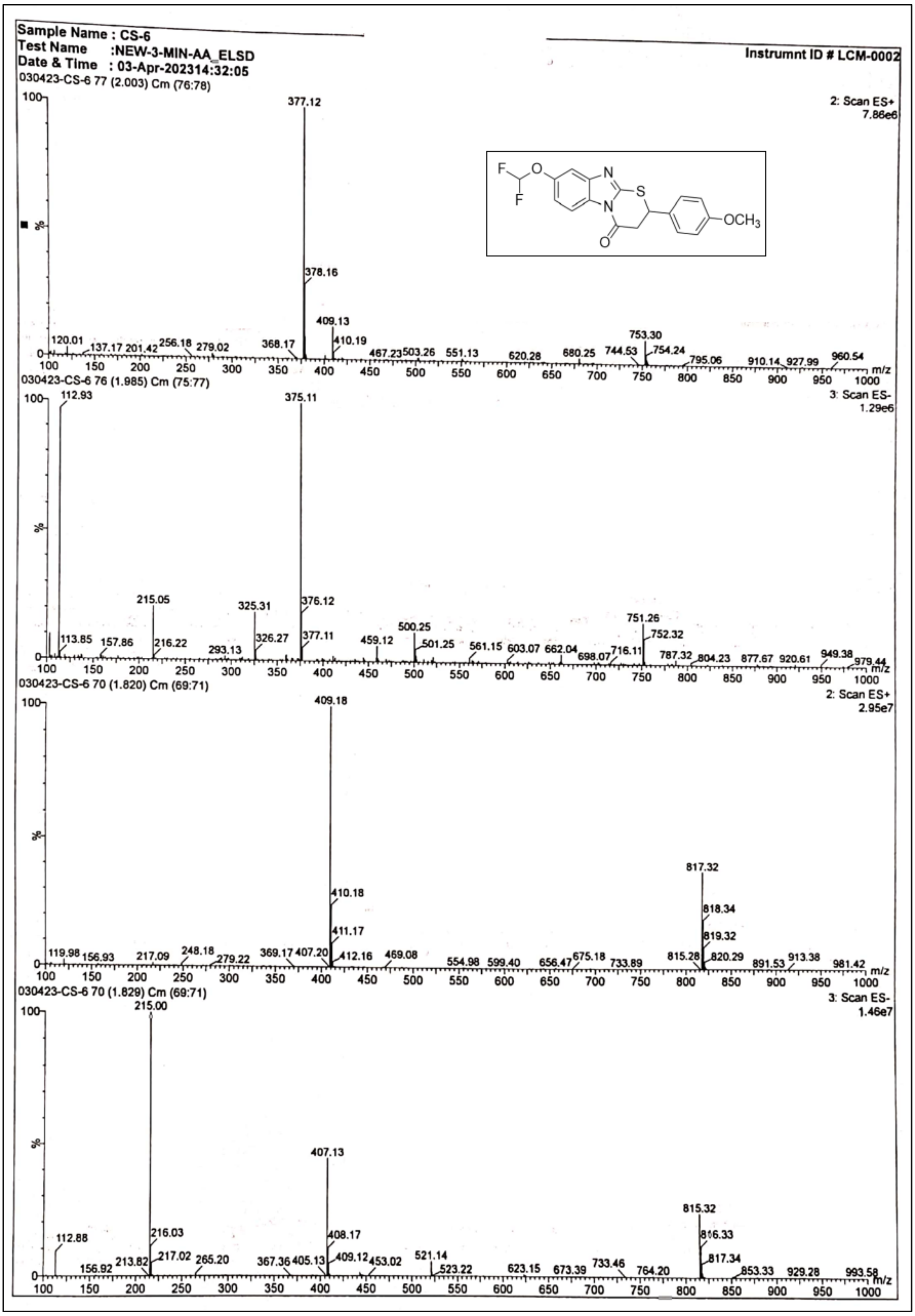

Figure S19: Compound CS6

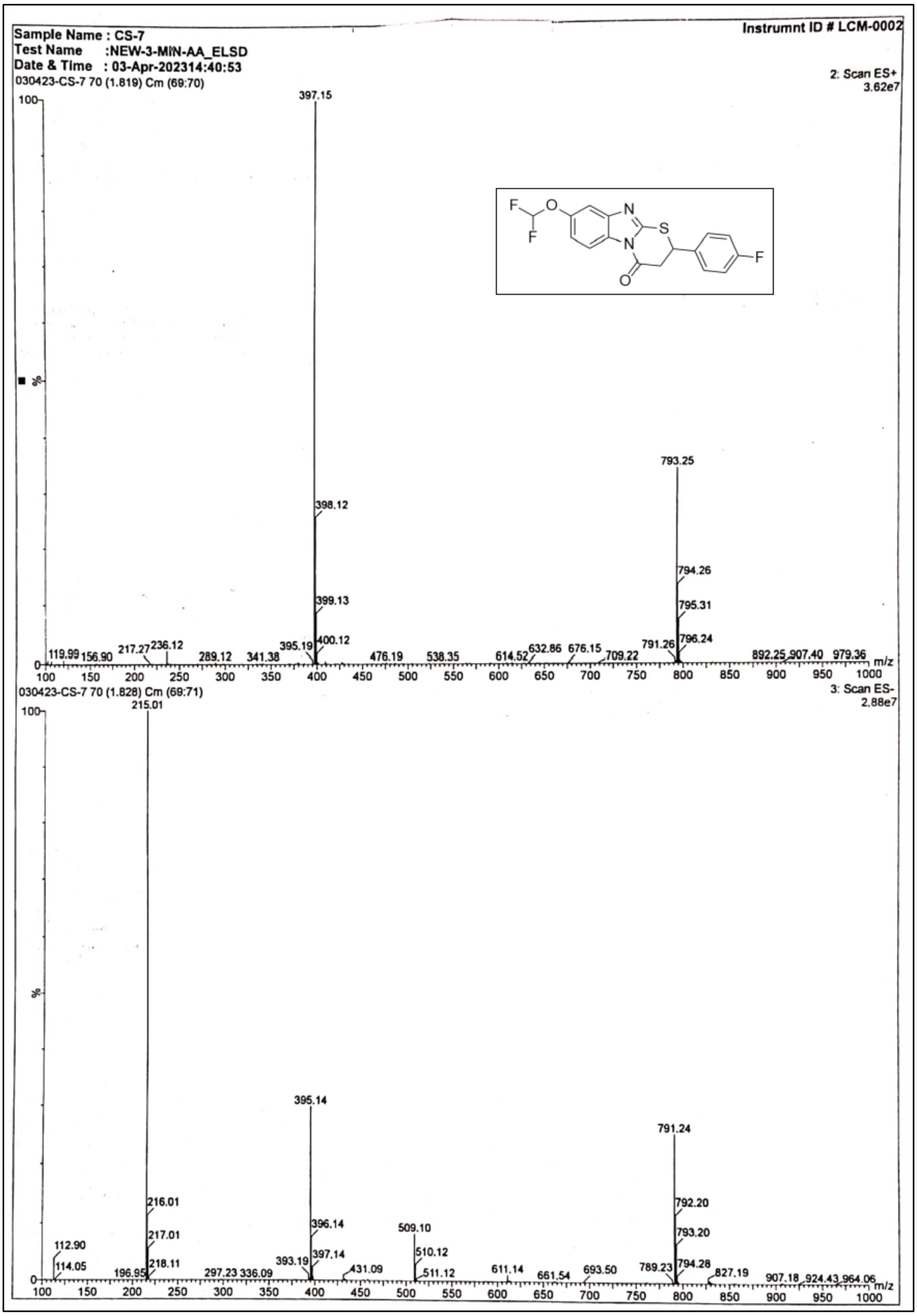

Figure S20: Compound CS7

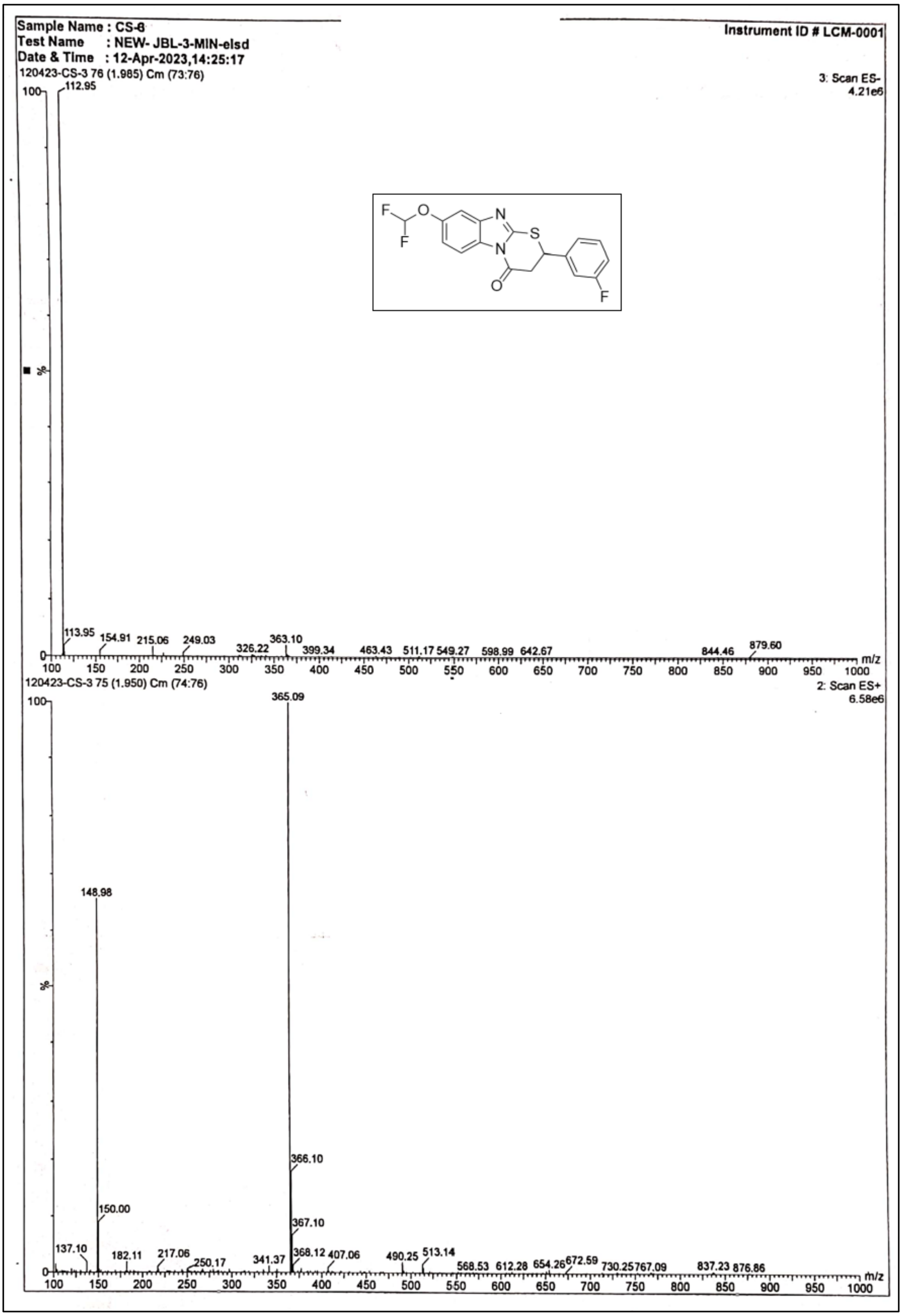

Figure S21: Compound CS8

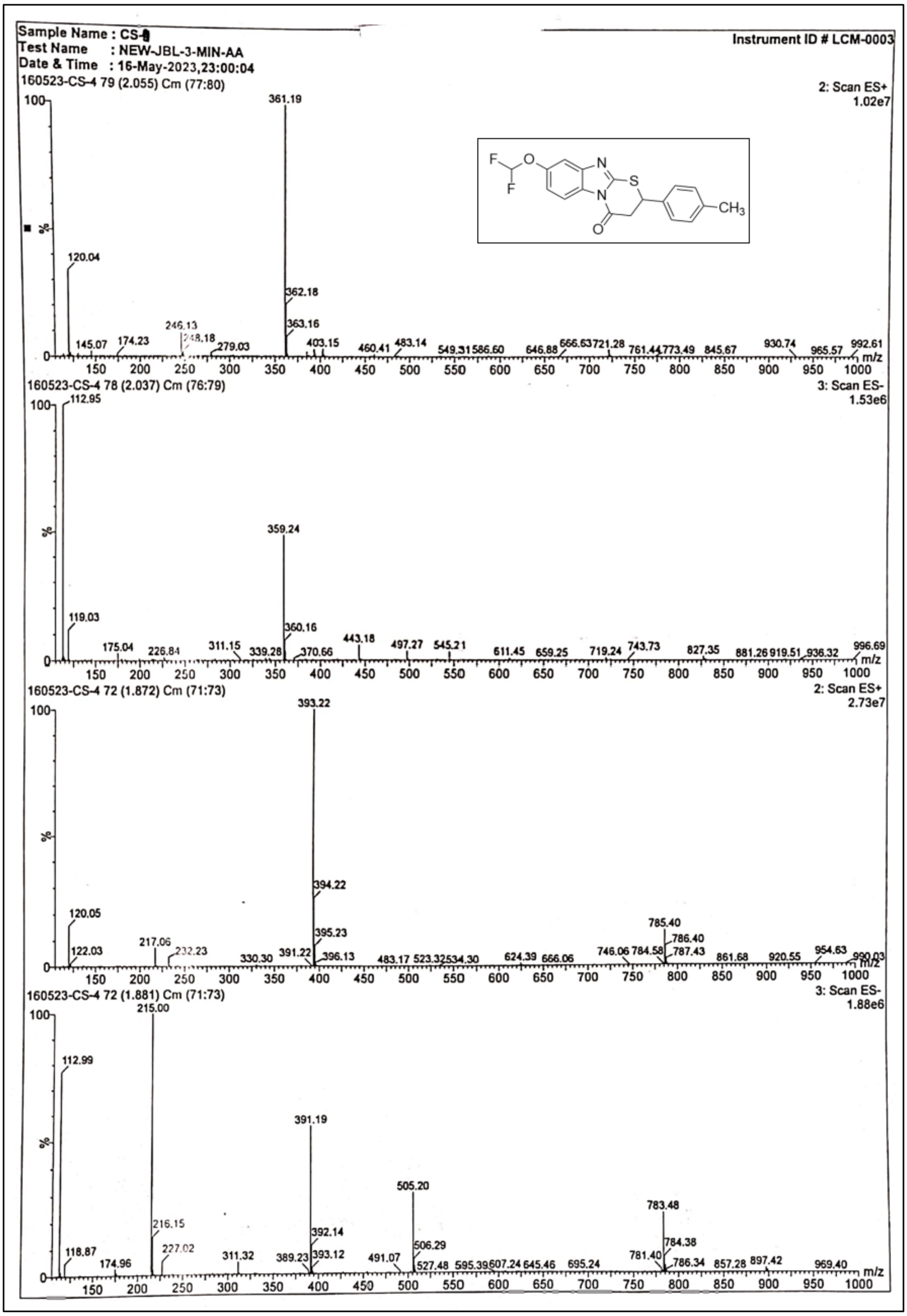

Figure S22: Compound CS9

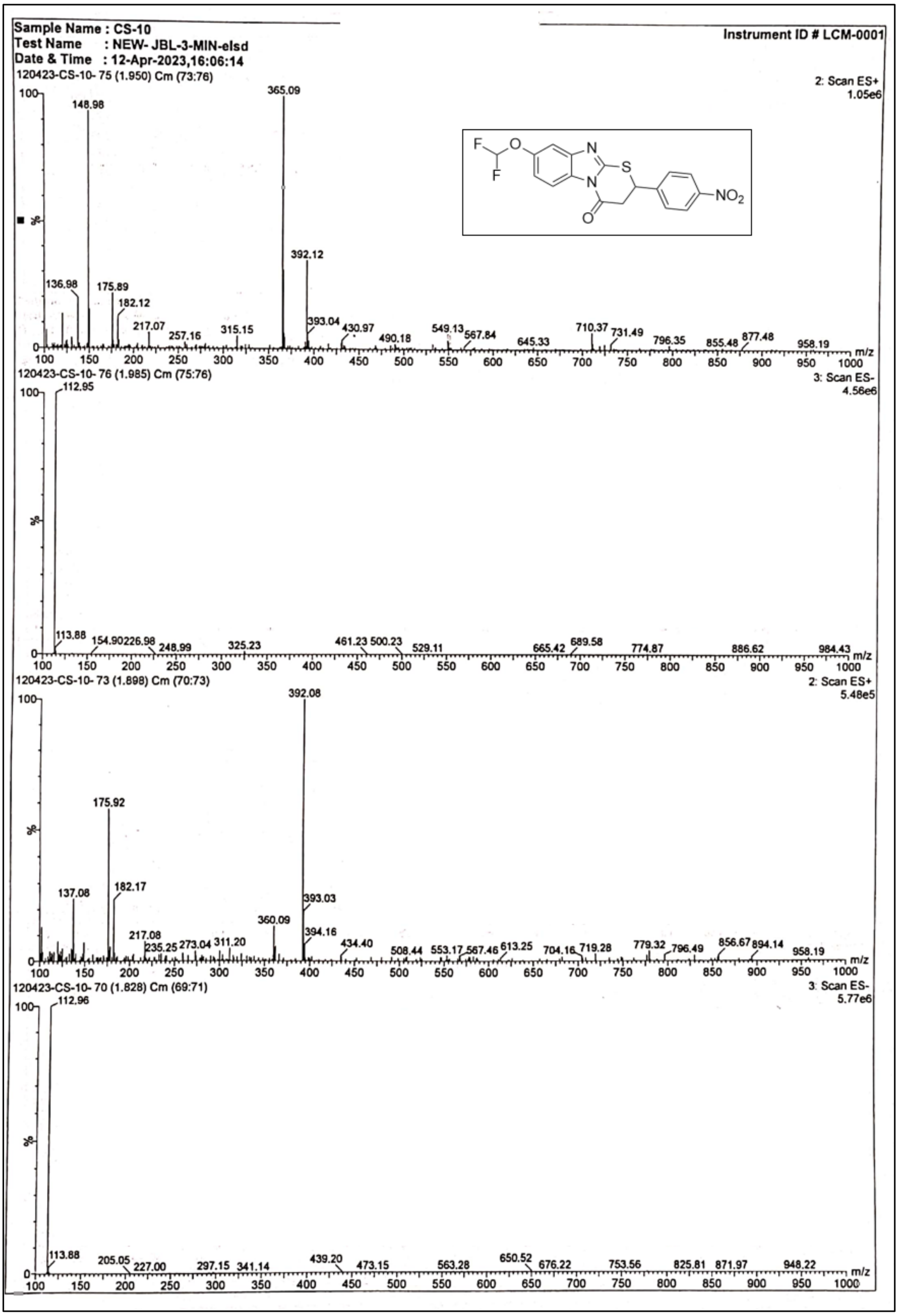

Figure S23: Compound CS10
